# Integrative evaluation and experimental validation of the immune-modulating potential of dysregulated extracellular matrix genes in high-grade serous ovarian cancer prognosis

**DOI:** 10.1186/s12935-023-03061-y

**Published:** 2023-09-30

**Authors:** Qihui Wu, Xiaoyun He, Jiaxin Liu, Chunlin Ou, Yimin Li, Xiaodan Fu

**Affiliations:** 1 Department of Gynecology, Xiangya Hospital, Central South University, Changsha, 410008 China; 2 National Clinical Research Center for Geriatric Disorders, Xiangya Hospital, Changsha, 410008 China; 3 Departments of Ultrasound Imaging, Xiangya Hospital, Central South University, Changsha, 410008 Hunan China; 4 Department of Pathology, School of Basic Medical Sciences, Central South University, Changsha, 410078 China; 5 Department of Pathology, Xiangya Hospital, Central South University, No.87 Xiangya Road, Changsha, 410008 China; 6 Department of Pathology, Ruijin Hospital, Shanghai Jiaotong University School of Medicine, No. 197, Ruijin Er Road, Huangpu District, Shanghai, 200025 China

**Keywords:** Extracellular matrix, Immunity, Prognosis, Treatment, Ovarian carcinoma

## Abstract

**Background:**

High-grade serous ovarian cancer (HGSOC) is a challenging malignancy characterized by complex interactions between tumor cells and the surrounding microenvironment. Understanding the immune landscape of HGSOC, particularly the role of the extracellular matrix (ECM), is crucial for improving prognosis and guiding therapeutic interventions.

**Methods and results:**

Using univariate Cox regression analysis, we identified 71 ECM genes associated with prognosis in seven HGSOC populations. The ECMscore signature, consisting of 14 genes, was validated using Cox proportional hazards regression with a lasso penalty. Cox regression analyses demonstrated that ECMscore is an excellent indicator for prognostic classification in prevalent malignancies, including HGSOC. Moreover, patients with higher ECMscores exhibited more active stromal and carcinogenic activation pathways, including apical surface signaling, Notch signaling, apical junctions, Wnt signaling, epithelial-mesenchymal transition, TGF-beta signaling, and angiogenesis. In contrast, patients with relatively low ECMscores showed more active immune-related pathways, such as interferon alpha response, interferon-gamma response, and inflammatory response. The relationship between the ECMscore and genomic anomalies was further examined. Additionally, the correlation between ECMscore and immune microenvironment components and signals in HGSOC was examined in greater detail. Moreover, the expression of MGP, COL8A2, and PAPPA and its correlation with FAP were validated using qRT-PCR on samples from HGSOC. The utility of ECMscore in predicting the prospective clinical success of immunotherapy and its potential in guiding the selection of chemotherapeutic agents were also explored. Similar results were obtained from pan-cancer research.

**Conclusion:**

The comprehensive evaluation of the ECM may help identify immune activation and assist patients in HGSOC and even pan-cancer in receiving proper therapy.

**Supplementary Information:**

The online version contains supplementary material available at 10.1186/s12935-023-03061-y.

## Introduction

Ovarian cancer stands as the most aggressive and lethal gynecological malignancy and ranks fifth among the leading causes of cancer-related deaths in developed countries [1, 2]. According to the most recent global cancer data, approximately 314,000 new cases of ovarian cancer were reported in 2020, with more than 200,000 deaths [1]. In the United States, approximately 20,000 new cases and 13,000 deaths are estimated in 2022 [2]. Ovarian cancer is classified based on genetic, histological, and tissue origin heterogeneity [3]. Remarkably, 70–80% of ovarian cancer-related deaths are attributed to high-grade serous ovarian carcinoma (HGSOC) [4]. Despite advancements in life expectancy for numerous solid tumors over the past decade, survival rates for HGSOC patients have remained relatively stagnant since the introduction of platinum-based therapy over four decades ago. And the 5-year overall survival rate for ovarian cancer has seen marginal improvement since 1980s [5]. This high mortality rate associated with HGSOC is primarily due to its frequent diagnosis at advanced stages, accompanied by susceptibility to relapse and drug resistance following first-line treatment [6]. Therefore, effective stratification of disease recurrence risk holds immense clinical value, facilitating early intervention and patient monitoring. Achieving this necessitates an in-depth grasp of the molecular mechanisms governing HGSOC progression, as these mechanisms could influence risk classification and novel therapeutic approaches.

Recent decades have unveiled the pivotal role of the tumor microenvironment (TME) in governing tumor clonal evolution and cancer cell responsiveness to chemotherapy and immunotherapy [7]. Within this context, the extracellular matrix (ECM), a major component of the TME, emerges as a pivotal regulator of cellular function [8]. Growing evidence suggests that extracellular signals originating from the TME can surpass cancer cell intrinsic signaling and influence tumor growth independently of tumor clonal heterogeneity [9, 10]. ECM, undergoing modification by cancer cells, stromal elements, and immune cells, has been shown to enhance cancer cell proliferation, survival, and metastasis [11–14]. The deregulation of ECM components has also been connected to HGSOC development and progression [15–20]. However, it is currently unknown how the ECM environment of HGSOC differs from the normal ovarian environment and whether ECM modifications can offer diagnostic and therapeutic insights for this poor-prognosis cancer. Furthermore, it’s crucial to recognize that matrix components function not as isolated entities but as an interconnected network of matrix molecules [21]. Recent research has established the prognostic and predictive value of dysregulated stromal components in early non-small cell lung cancer [22, 23]. A thorough exploration of how these matrix networks undergo modification in HGSOC tumors and their subsequent impact on cancer cell behavior warrants comprehensive exploration.

Immunotherapy utilizing immune checkpoint inhibitors (ICI) has revolutionized the cancer treatment paradigm by harnessing the immune system’s capabilities. However, clinical trials indicate limited efficacy of ICI immunotherapy in HGSOC [24]. This could be attributed to intricate tumor evasion mechanisms inherent to HGSOC. Notably, ECM is believed to play a pivotal role in immune pathway modulation [25, 26]. An in-depth analysis of the ECM’s involvement in HGSOC may provide novel insights into prognosis and therapeutic options.

In this study, we meticulously examined seven cohorts to identify 71 ECM genes associated with HGSOC prognosis. Based on these findings, we formulated an ECMscore signature comprised of 14 genes, demonstrating its potential as an independent predictor of overall survival across four cohorts. Notably, ECMscore emerged as a robust prognostic factor across prevalent malignancies, including bladder urothelial carcinoma, colon adenocarcinoma, pancreatic adenocarcinoma, and others. Subsequently, individuals with HGSOC were separated into two groups based on their ECMscores, and the association between their biological behaviors and gene alterations was further examined. The high ECMscore group correlated with stromal and carcinogenic pathways, while low ECMscore group displayed enrichment of immune-related pathways. We further scrutinized the interplay between ECMscore and the immunological landscape and conducted experiments elucidating the connection between ECMscore and cancer-associated fibroblasts (CAFs). Additionally, we discovered that ECMscore can reflect the immunological landscape of pan-cancer. In addition, we discovered that ECMscore can reflect the immunological landscape of pan-cancer. Furthermore, the impact of ECMscore in immunotherapy and chemotherapy was examined in an effort to identify a more scientific hierarchical management strategy that might provide guidance for HGSOC and possibly pan-cancer treatment.

## Materials and methods

### Dataset source and preprocessing

**Fig. 1:**
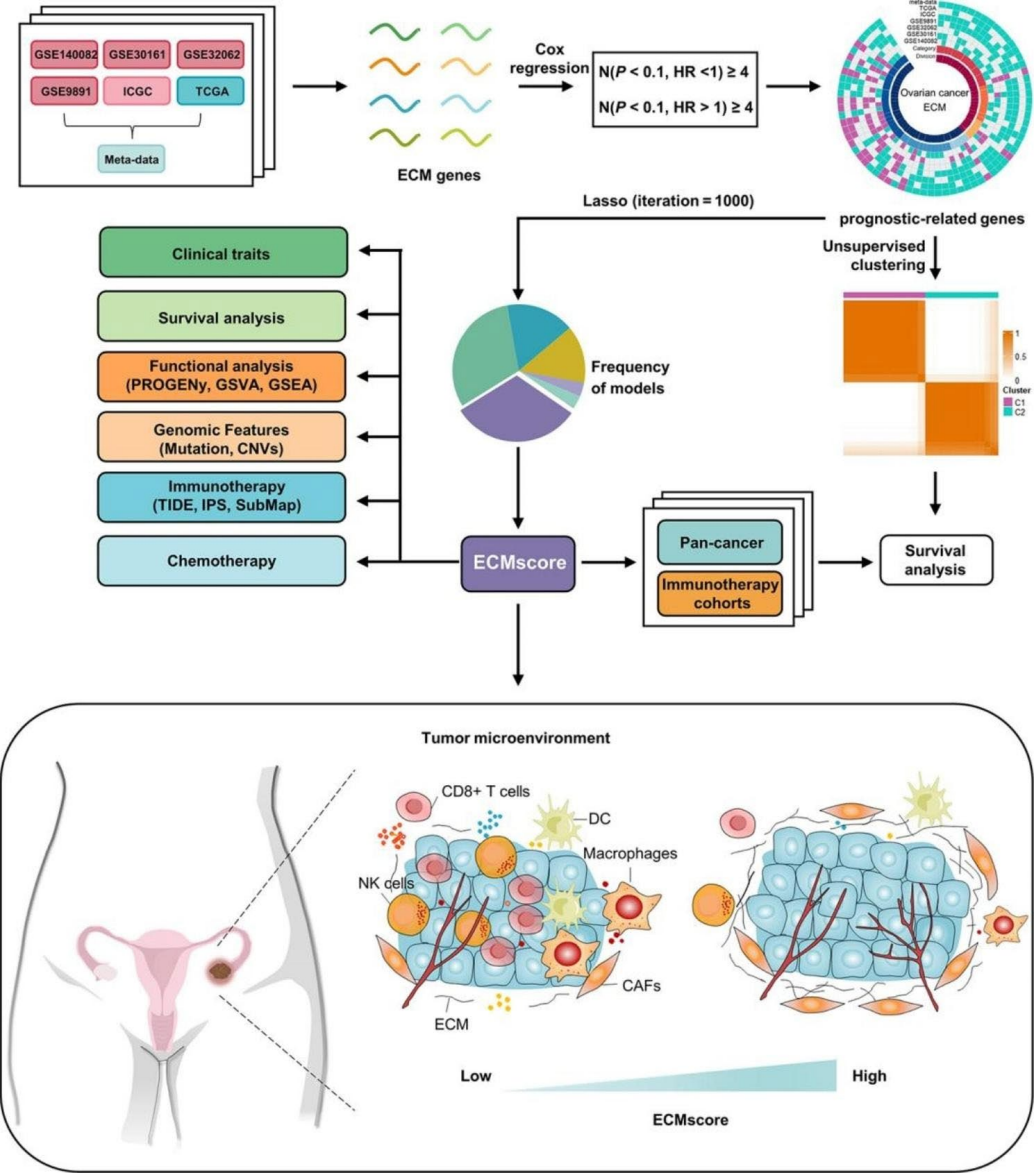
**Flow chart of this study.** The study begins with the identification of significantly correlated extracellular matrix (ECM) genes. Consensus clustering is employed to classify prognostic-related ECM genes into distinct extracellular matrix patterns, followed by an analysis of the association between different ECM clusters and patient prognosis. Subsequently, a LASSO algorithm is applied to construct an ECMscore signature. The risk model is comprehensively evaluated using clinical characteristics, survival analysis, functional enrichment annotations, genomic features, and the potential for predicting immunotherapy and chemotherapy responses. Finally, the prognostic relevance of the model is further validated in pan-cancer analysis and immunotherapy assessment

The overall design of this study is depicted in Fig. 1.

We gathered data from six distinct transcriptomic cohorts of HGSOC patients, encompassing four microarray cohorts from the Gene Expression Omnibus (GEO: GSE140082, GSE30161, GSE32062, and GSE9891), one RNA-sequencing cohort from the International Cancer Genome Consortium (ICGC: OV-AU), and another RNA-sequencing cohort from The Cancer Genome Atlas (TCGA-OV). Corresponding clinical data were also collected, and individuals with missing overall survival (OS) values and those with a survival duration of less than one month were excluded from the analysis. Detailed baseline information for all six HGSOC datasets is outlined in Supplementary Table 1. To facilitate the comparison of ECM gene expression between normal ovarian tissue and ovarian cancer patients, we utilized the data from GSE18520 and GSE40595 (Supplementary Table 1). Pan-cancer statistics and related clinical data were extracted from the TCGA database. In addition, we retrospectively evaluated four distinct transcriptomic cohorts for immunotherapy. The IMvigor210 cohort was retrieved from an online database (http://researchpub.gene.com/IMvigor210CoreBiologies). Furthermore, two separate microarray datasets, GSE100797 and GSE35640, were acquired from the GEO database. Nathanson’s cohort’s clinical and expression profiling data were obtained from published studies [27]. We transformed the RNA-sequencing data into more comparable transcripts per kilobase million (TPM) values for the RNA-sequencing cohorts. The meta-data consolidation was carried out using the “ComBat” algorithm from the “sva” package for the four GEO cohorts, ICGC-OV, and TCGA-OV datasets. A complete list of human ECM genes was obtained from the website (http://matrisomeproject.mit.edu/) [28, 29], which encompassed 274 core matrisome genes and 753 matrisome-associated genes (Supplementary Table 2).

### Genomic alterations analysis

The “TCGAbiolinks” software was utilized to retrieve from TCGA somatic mutations and copy number alterations (CNAs) corresponding to HGSOC samples with RNA-sequencing data. The “maftools” and “ComplexHeatmap” packages were used to analyze and visualize somatic variations for somatic mutation data. The “GISTIC 2.0” module of the GenePattern website (https://genepattern.org) was utilized to identify ECM gene amplification (GISTIC value ≥ 1) and deletion (GISTIC value ≤ -1).

### Human tissue specimens

We recruited 30 HGSOC patients who had undergone curative resection at Xiangya Hospital, Central South University (Supplementary Table 3). The informed consent form was signed by all patients enrolled in the study. The Xiangya Hospital Ethics Committee authorized this study.

### RNA extraction and quantitative real-time polymerase chain reaction (qRT-PCR)

Total RNA was extracted using FFPE RNA Extraction Kits (AmoyDx, Xiamen, China) following the manufacturer’s instructions. RNA purity and quantity were assessed using the NanoDrop 1000 Spectrophotometer (Thermo Fisher, USA), with OD260/OD280 ratios of 1.8-2.0 and OD260/230 ratios of 2.0-2.2. Reverse transcription of the first-strand cDNA was performed using HiScript II Reverse Transcriptase (Vazyme, Nanjing, China) with 1 µg of total RNA. Quantitative real-time PCR (qRT-PCR) was conducted on an ABI Prism 700 thermal cycler (Applied Biosystems, Foster City, CA, USA) as described previously [30]. GAPDH was used as the normalizer for RNA quantification. Each experiment was performed in triplicate. The primer sequences are is outlined in Supplementary Table 4.

### Consensus clustering

Consensus clustering, an unsupervised class discovery tool, was used to identify ECM clusters using the “ConsensusClusterPlus” R package [31]. The k value was adjusted to be between 2 and 6, with 1,000 initial resamples conducted for the clustering process. Here, the k value represents the number of clusters. The consensus matrix (CM) and cumulative distribution function (CDF) were utilized to determine the optimal k value or the most suitable number of clusters. Based on the expression levels of prognostic-related ECM genes across different cohorts, we subsequently identified two distinct ECM clusters.

### Generation of the ECMscore

Univariate Cox regression analysis was employed to identify ECM genes with predictive potential in the six separate cohorts and the meta-data. Considering the stringent demands of multiple testing correction and the challenges posed by limited sample sizes in specific cohorts, which could potentially overlook latent ECM genes with prognostic significance, we implemented a screening process to identify genes that satisfied both criteria: having a *P* value < 0.1 and demonstrating consistent hazard ratio (HR) direction across over four cohorts (Supplementary Table 5). The Lasso method, a form of shrinkage estimation, was employed to create a refined model by constructing a penalty function that shrinks specific coefficients while setting others to zero. Further compression of variables was achieved through Lasso regression to reduce the number of genes in the risk model. The method employed to derive the ECMscore was similar to prior studies [32, 33]. The set of 71 ECM genes underwent Cox proportional hazards regression with tenfold cross-validation using the ‘glmnet’ package. After 1000 iterations, a total of 7 signals were generated. The signal with the highest frequency was selected as the optimal signal for subsequent investigations. Consequently, we employed the 14 ECM-related genes within this model to formulate our ECMscore signature.:

$$\text{E}\text{C}\text{M}\text{s}\text{c}\text{o}\text{r}\text{e}={\sum }_{i=1}^{n}\text{C}\text{o}\text{e}\text{f}\text{i}\text{*}{x}_{i}$$

Where n was the number of prognostic genes, ${x}_{i}$ was the expression value of each prognostic gene, and Coefi was the regression coefficient of each prognostic gene in the LASSO algorithm. Median risk scores from different HGSOC patient cohorts were used to categorize patients into low- and high-ECMscore groups. Moreover, the prognostic performance of the ECMscore was assessed using Kaplan-Meier curves, time-dependent area under the receiver operating characteristic curve (AUC), and the concordance (c)-index.

### Functional and pathway enrichment analyses

The ‘‘clusterProfiler’’ package was used to examine prognostic-related ECM genes in the Kyoto Encyclopedia of Genes and Genomes (KEGG) pathways and Gene Ontology (GO) terms [34]. To investigate the malignant signaling pathway activities of HGSOC patients, we applied the “PROGENy” package, a method that can deduce pathway activity from gene expression by employing core pathway-responsive genes [35]. The “clusterProfiler” and “GSVA” packages were subsequently used to undertake a gene set enrichment analysis (GSEA) study and single sample GSEA (ssGSEA) analysis with the objective of discovering underlying biological functions among distinct ECMscore groups [36]. The gene sets “h.all.v7.5.1.symbols”, “c5.go.mf.v7.5.1.symbols”, “c5.go.bp.v7.5.1.symbols”, and “c2.cp.kegg.v7.5.1.symbols” from the MSigDB database were chosen as the reference gene sets.

### Evaluation of the immunogenomic landscape

The infiltration levels of 28 immune cells and the activation of the immune pathway for each HGSOC patient were determined using “ssGSEA” and the “GSVA” software package [37]. By utilizing the “ESTIMATE” program, the immunological score, ESTIMATE score, stromal score, and tumor purity were calculated [38, 39]. CIBERSORT, CIBERSORT-ABS, EPIC, TIMER, QUANTISEQ, MCPCOUNTER, and xCELL were used to confirm immune cell infiltrations [38, 40–44]. T cell–inflamed cytolytic activity (CYT) and gene expression profile (GEP) were determined using previously described methods [45, 46]. Tracking Tumor Immunophenotype (TIP; http://biocc.hrbmu.edu.cn/TIP/index.jsp) was used to obtain the activity scores of the cancer immunity cycle in HGSOC patients [47].

### Immunotherapy and chemotherapy response prediction

The tumor immune dysfunction and exclusion (TIDE), subclass mapping (SubMap) algorithms, and immunophenoscore (IPS) were used to predict responses of HGSOC patients to immune checkpoint blockade (ICB) as previously documented [48–51]. To study the sensitivity difference of medications between low- and high-ECMscore groups, we used the “pRRophetic” software to assess the half-maximal inhibitory concentration (IC_50_) of drugs of each HGSOC patient by ridge regression using the Genomics of Drug Sensitivity in Cancer (GDSC; https://www.cancerrxgene.org) database [52]. *P* values < 0.05 were statistically significant, while lower IC_50_ values indicated higher sensitivity. To discover prospective therapeutic agents in distinct groups, a spearman’s correlation analysis was conducted between IC_50_ values and ECMscore, yielding nine medications with |Spearman’s R| ≥ 0.30.

### Statistical analysis

The R software (https://www.r-project.org/, version 4.0.5) was used for statistical analysis and graph visualization. For normally distributed variables, differences between two groups were assessed using Student’s t-test, while comparisons involving more than two groups employed the one-way ANOVA test. In cases of non-normally distributed variables, comparisons between two groups utilized the Wilcoxon rank-sum test, whereas comparisons among more than two groups employed the Kruskal-Wallis test. Furthermore, categorical variable comparisons between groups were conducted using either the chi-square test or Fisher’s exact test, contingent upon sample size and expected cell frequencies. Correlation analysis between two continuous variables entailed Pearson’s correlation for normally distributed data with linear relationships, while Spearman’s correlation was applied for non-normally distributed data or data exhibiting monotonic relationships. Kaplan-Meier curves and the log-rank test were utilized to compare the OS or PFS of various ECMscore groups. Using univariate and multivariate Cox regression models, the prognostic factors were evaluated. Using the “timeROC” package, the time-dependent AUC was determined. A two-sided *P* value < 0.05 was considered statistically significant (*P < 0.05 **P < 0.01, ***P < 0.001, ****P < 0.0001).

## Results

### Identification of Prognostic-Associated ECM genes in HGSOC Patients

**Fig. 2:**
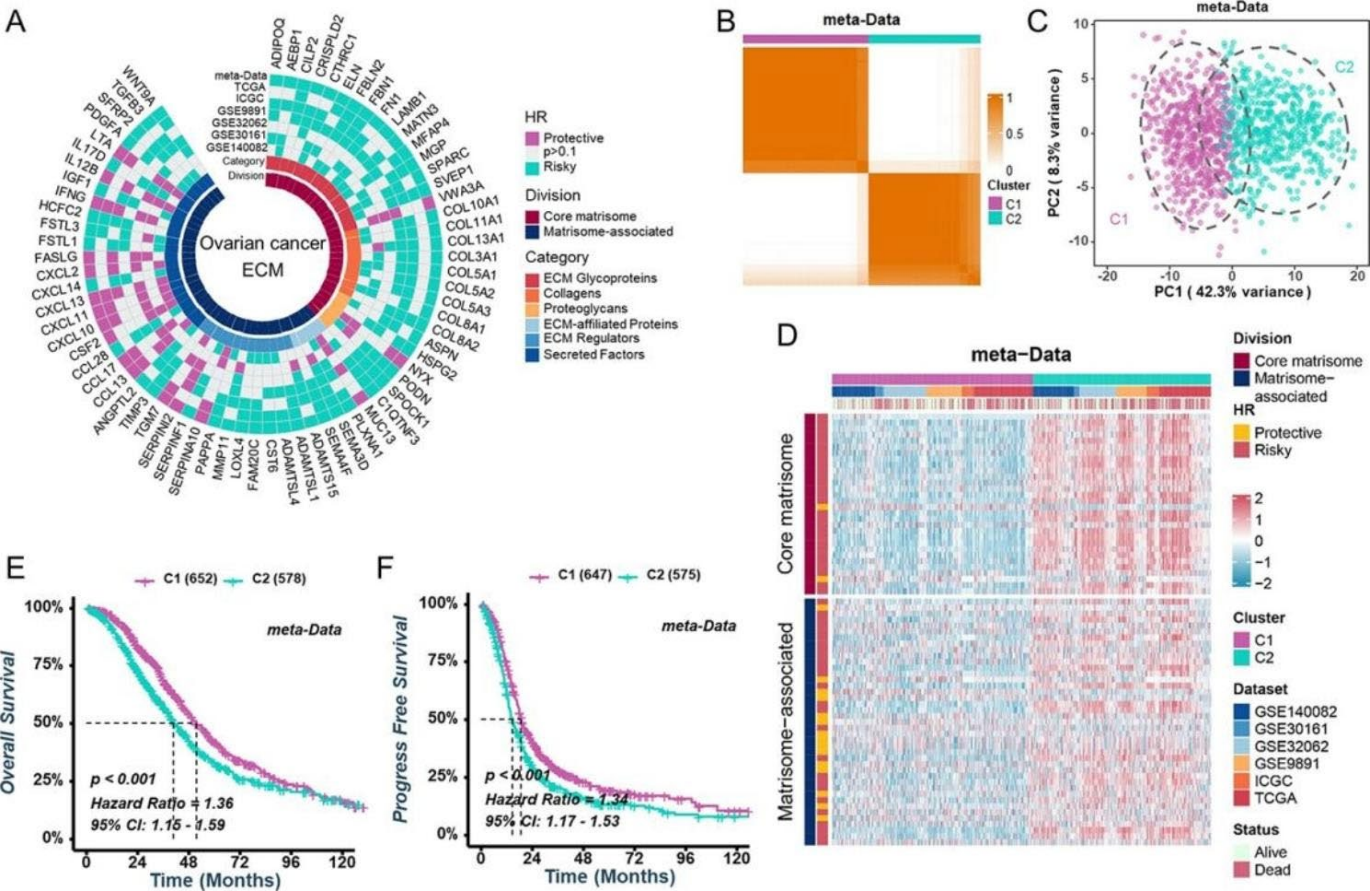
**Identification of two ECM clusters in HGSOC patients.** (**A**) Univariate Cox regression analysis used to identify ECM genes associated with OS across various cohorts. (**B**) Cumulative distribution function of consensus clustering for k = 2 in the meta-data cohort. (**C**) Principal component analysis demonstrating the separation between cluster C1 and cluster C2 based on prognostic-related ECM genes. (**D**) Heatmap illustrating the expression profiles of prognostic-related ECM genes in the meta-data cohort. (**E**, **F**) Kaplan-Meier analysis depicting the OS (**E**) and PFS (**F**) differences between different ECM clusters in the meta-data cohort

In order to gain a comprehensive understanding of the underlying mechanisms involving ECM genes in HGSOC, we assembled a total of 1230 HGSOC patients (meta-data) and addressed batch effects using the “ComBat” algorithm (Supplementary Fig. 1A, 1B). For the identification of ECM genes linked with prognosis, we initiated univariate Cox regression analysis across seven cohorts. This analysis revealed a set of 71 ECM genes, encompassing 30 core matrisomal genes and 41 matrisome-related genes, significantly associated with OS in HGSOC (Fig. 2A). We compared the mRNA expression levels of these prognostic-related genes in normal ovarian and HGSOC samples from the GSE18520 and GSE40595 datasets. Among these genes, 11 showed elevated expression in HGSOC samples, while 5 exhibited downregulated expression (Supplementary Fig. S1C). We further evaluated the methylation level and the copy number variation (CNV) modifications of 71 ECM genes in ovarian cancer, discovering potential associations between methylation changes, CNV alterations, and abnormal ECM gene expression (Supplementary Fig. S1D, S1E). In addition, the incidence of somatic mutations in 71 ECM genes was studied in HGSOC. The top 20 mutations of prognostic-related ECM genes were displayed, with COL5A3 (7%) showing the highest mutation frequency, followed by HSPG2 (6%) and LAMB1 (6%) (Supplementary Fig. S1F). To provide insight into their biological roles, GO and KEGG analyses revealed that these prognostic-related ECM genes were enriched for extracellular matrix organization, collagen-containing extracellular matrix, extracellular matrix structural component, and cytokine-cytokine receptor interaction (Supplementary Fig. S1G, S1H). The correlation analysis revealed that certain ECM genes in the TCGA cohort and meta-data were substantially interrelated (Supplementary Fig. S2A).

### Distinct ECM modification patterns in HGSOC patients

Based on the expression profiles of these 71 prognostic-related ECM genes, an unsupervised consensus clustering analysis was conducted to identify the specific ECM modification patterns of HGSOC patients, and two distinct modification patterns were revealed in metadata (Fig. 2B and Supplementary Fig. S3A). The principal component analysis confirmed that the expression levels of the 71 ECM genes also separate the two groups (Fig. 2C). As demonstrated in Fig. 2D, cluster 2 (C2) tended to have higher ECM gene expression than cluster 1 (C1). The Kaplan-Meier survival study indicated that HGSOC patients in C2 had worse OS and progression-free survival (PFS) than in C1, suggesting that this classifier was a prognostic predictor (Fig. 2E F). Using the same clustering methodology, patients with HGSOC from various cohorts were also classified into two subgroups (Supplementary Fig. S3A, S3B). Consistent with previous findings, the cluster with increased ECM gene expression had a much worse prognosis (Supplementary Fig. S4A-S4C).

### Identification and clinical validation of ECMscore as a prognostic biomarker

**Fig. 3:**
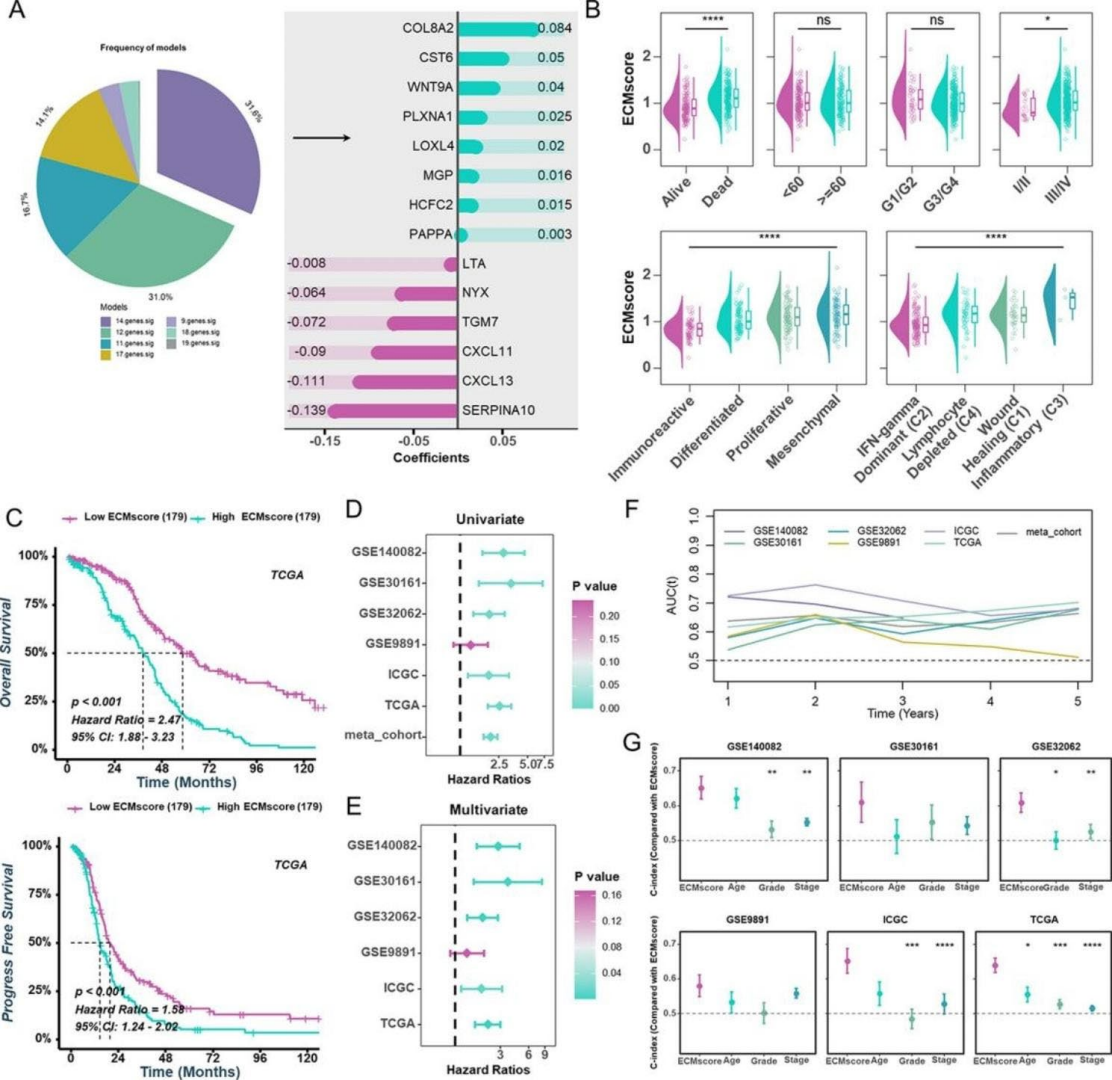
**Construction of a prognostic signature based on ECM genes.** (**A**) Frequency distribution of prognostic signatures after 1000 iterations (left pie chart) and regression coefficients of each gene in the ECMscore signature determined by the LASSO algorithm (right bar chart). (**B**) Associations between ECMscore and clinical features including survival status, age, grade, stage, TCGA molecular subtypes, and immune subtypes. (**C**) Kaplan-Meier analysis illustrating the differences in OS (upper) and PFS (bottom) between low- and high-ECMscore groups in the TCGA-OV cohort. (**D**) Univariate Cox regression analysis of ECMscore across various cohorts, with dashed line representing HR = 1. (**E**) Multivariate Cox regression analysis showing the effect of ECMscore and clinicopathological characteristics on survival in different cohorts. The HR value of ECMscore was adjusted for age, grade, and stage in various cohorts, with dashed line indicating HR = 1. (**F**) Time-dependent area AUC values of ECMscore in different cohorts. (**G**) C-index comparison of ECMscore with clinicopathological characteristics in different cohorts

Considering the significant interrelations among some ECM genes and aiming to determine the optimal prognostic biomarker for clinical application, the 71 prognostic-related ECM genes from the TCGA-OV cohort underwent Cox proportional hazards regression with lasso penalty to reduce the gene count within the risk model. After 1000 iterations, seven prognostic signatures were obtained, and a 14-gene signature named ECMscore was further screened out based on the highest frequency (Fig. 3A and Supplementary Table 6). These genes were derived from three core matrisomal genes and eleven matrisome-associated genes (Fig. 2A). Next, we calculated each patient’s ECMscore as follows: ECMscore = 0.084 * COL8A2 + 0.05 * CST6–0.09 * CXCL11–0.111 * CXCL13 + 0.015 * HCFC2 + 0.02 * LOXL4–0.008 * LTA + 0.016 * MGP − 0.064 * NYX + 0.003 * PAPPA + 0.025 * PLXNA1–0.139 * SERPINA10–0.072 * TGM7 + 0.04 * WNT9A (Fig. 3A). ECMscore was substantially correlated with survival status, stage, TCGA molecular subtypes, and immunological subtypes (Fig. 3B). Patients with HGSOC were split into low and high ECMscore groups using a median cutoff, and it was discovered that the high ECMscore group had significantly worse OS and PFS in different cohorts (Fig. 3C, Supplementary Fig. S5A and S5B). In five cohorts, univariate and multivariate Cox regression analysis revealed that the ECMscore served as an independent predictive predictor for OS (Fig. 3D and E). In the meantime, outcomes from the time-dependent AUC analyses indicated that ECMscore could predict HGSOC patient OS with compatible accuracy across multiple independent cohorts (Fig. 3F). In comparison to other clinical parameters (age, grade, and stage), ECMscore demonstrated significantly superior accuracy in GES140082, GSE32062, ICGC, and TCGA cohorts, while displaying comparable accuracy in the remaining two cohorts (Fig. 3G). Beyond HGSOC, we extended our exploration of ECMscore’s prognostic efficacy for OS and PFS to other malignancies. The results highlighted that ECMscore serves as a robust prognostic stratification predictor across the most prevalent malignancies (Supplementary Fig. S6A, S7A).

### Uncovering biological behavior and pathway associations of ECMscore groups

**Fig. 4:**
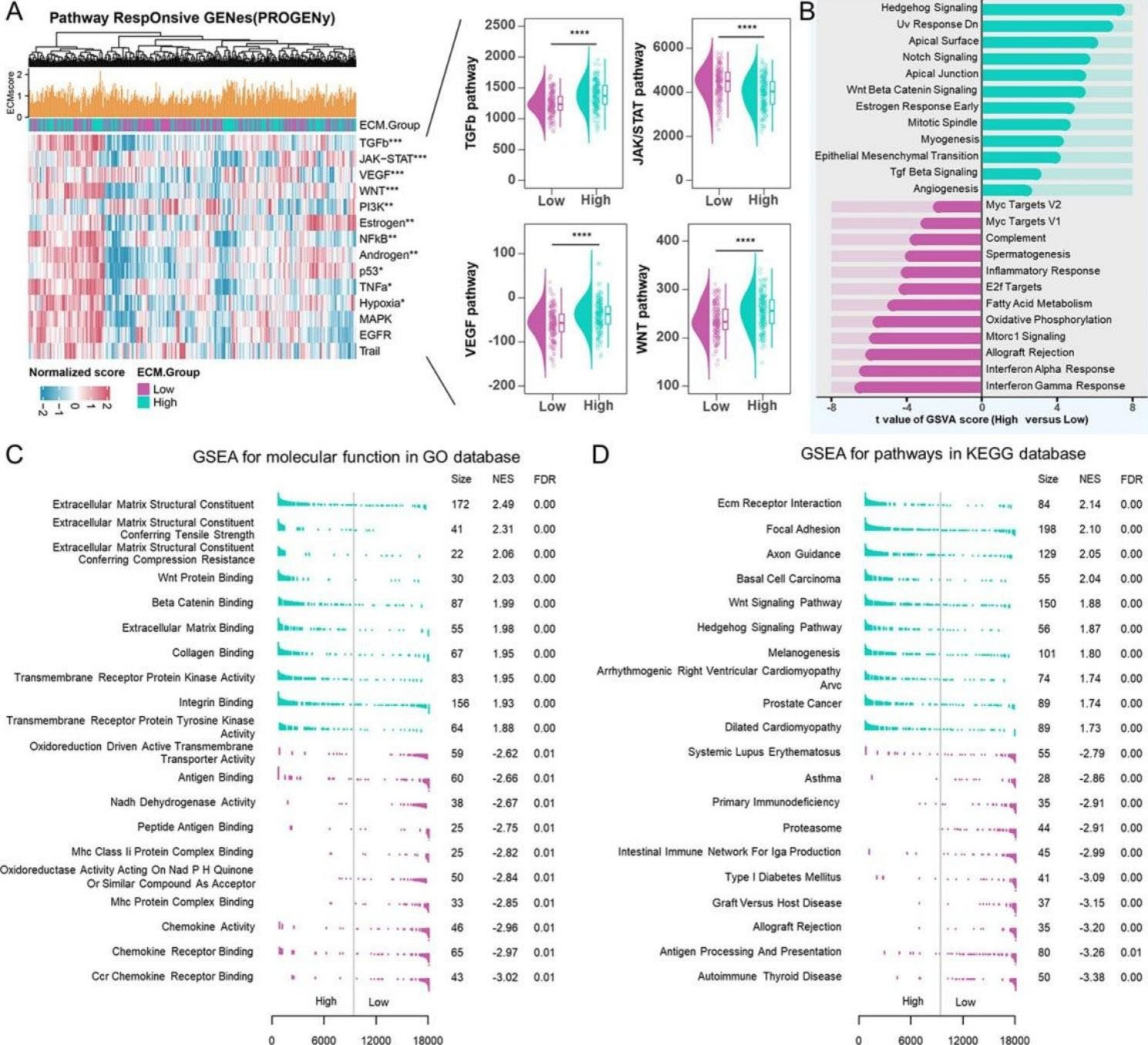
**Underlying biological functions of different ECMscore groups.** (A) Heatmaps (left) and box plot (right) illustrating the activity scores of signaling pathways in low- and high-ECMscore groups within the TCGA-OV cohort. (B) Bar graph showing the disparity in enrichment scores based on GSVA analysis between low- and high-ECMscore groups. (C, D) GSEA of biological processes from the GO database (C) and KEGG pathway gene sets (D). The top 10 positively and negatively associated pathways with ECMscore are presented

To investigate the underlying biological behavior between different ECMscore groups, we examined the relationship between ECMscore and the activities of malignant signaling pathways. Our analysis unveiled positive correlations between ECMscore and TGF-ß, VEGF, and WNT signaling pathways, while revealing a negative correlation with the JAK/STAT pathway (Fig. 4A). Subsequently, GSVA enrichment analysis showed that the high ECMscore group was characterized by stromal and carcinogenic activation pathways, including apical Surface, Notch signaling, apical Junction, Wnt signaling, epithelial-mesenchymal transition (EMT), TGF-ß signaling, and angiogenesis, thereby affirming earlier findings [53]. Conversely, the low ECMscore group exhibited enrichment in immune-related pathways such as interferon alpha response, interferon-gamma response, and inflammatory response (Fig. 4B). Comparable outcomes were achieved through GSEA analyses of GO annotation and KEGG pathways (Fig. 4C and D, and Supplementary Fig. S8A). These findings collectively imply an association between the ECMscore and the immune landscape of ovarian cancer.

### Exploring genomic characteristics of different ECMscore groups

**Fig. 5:**
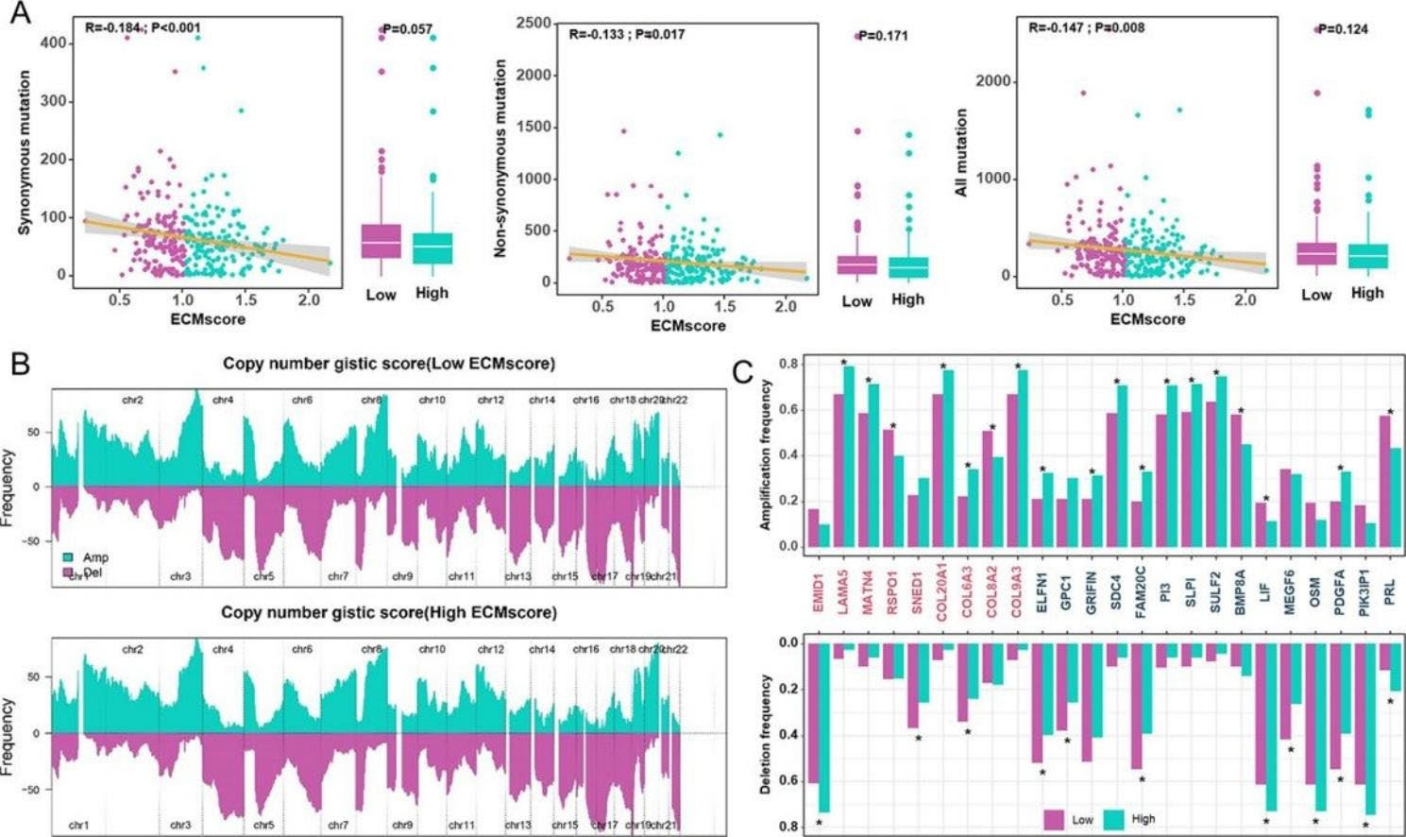
**Genomic Features of different ECMscore groups.** (**A**) Distribution and relationship between total mutation counts, synonymous mutation counts, non-synonymous mutation counts, and ECMscore, depicted through boxplots and scatterplots. The distributions of these features in low and high ECMscore groups are shown. (**B**) Comparison of CNV through genomic amplifications and deletions between low- and high-ECMscore groups. (**C**) Chromosomal locations of ECM genes exhibiting significant amplifications and deletions in broad regions of copy number alterations

Genome aberrations, such as somatic mutations and copy number alterations, play a crucial role in the development of HGSOC. To investigate ECMscore-related pathways in HGSOC, we investigated somatic mutations within distinct ECMscore groups. The results indicated a weak negative correlation between ECMscore and both synonymous and nonsynonymous somatic mutations. However, there were no statistically significant differences in somatic mutations between high and low ECMscore groups (Fig. 5A). Among the top 20 genes with the highest mutation frequencies, only DNAH5 exhibited differential mutations between the two groups (Supplementary Fig. S9A). As shown in Supplementary Fig. S9B, genes with different frequencies between the low and high ECMscore groups were displayed using Fisher’s exact test with a *P* value threshold of < 0.05. Subsequently, we examined CNV between different ECMscore groups. Both low- and high-risk groups displayed genomic amplifications and deletions (Fig. 5B). Nonetheless, only a limited number of chromosomal band-level discrepancies were observed when comparing the two groups (Supplementary Fig. S9C). Within these chromosomal bands, several ECM genes exhibited extensive amplifications in the high ECMscore group or deletions in the low ECMscore group (Fig. 5C).

### Unveiling the Nexus of ECMscore and the immune landscape

**Fig. 6:**
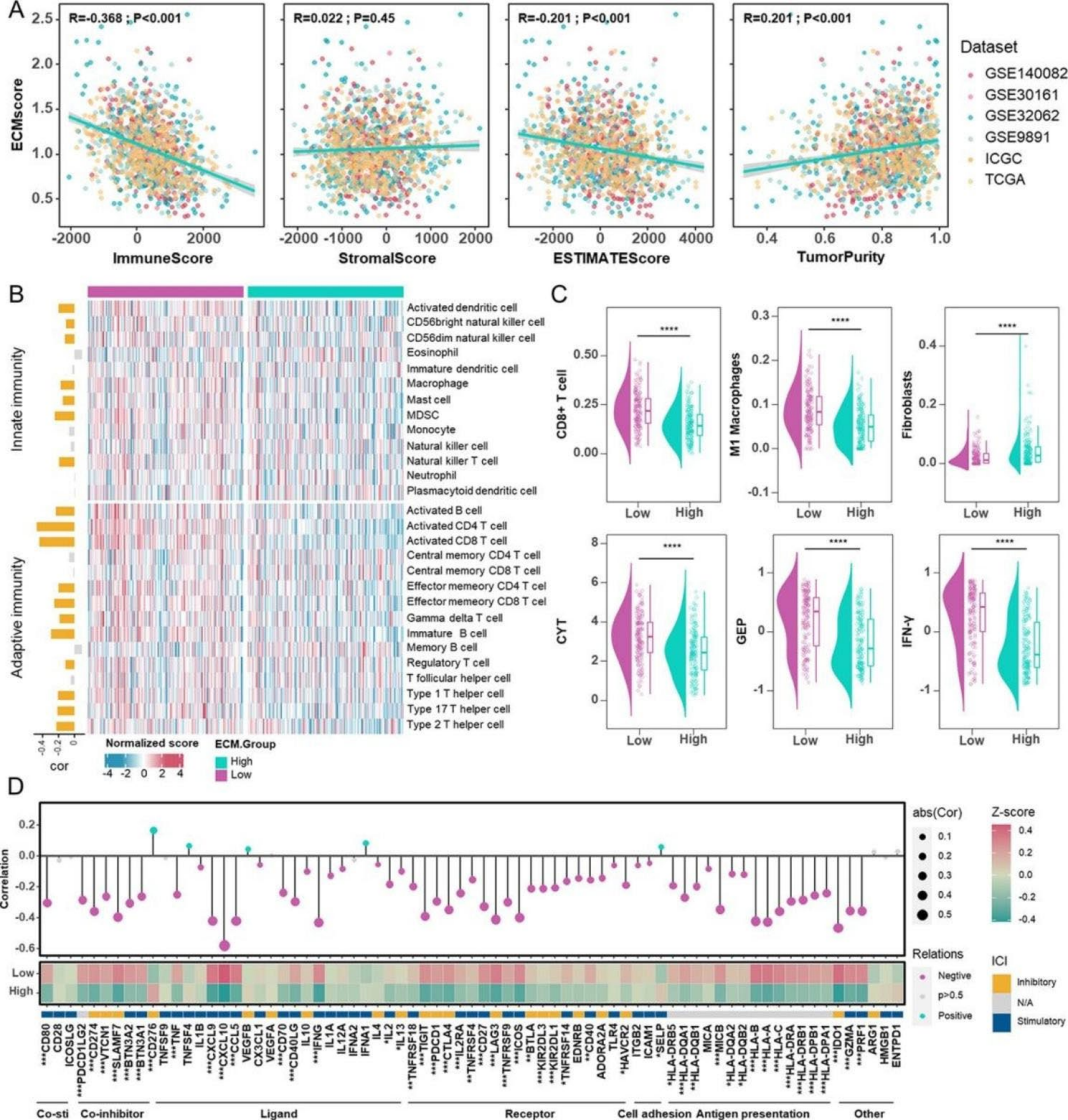
**Immune Landscape of different ECMscore groups.** (**A**) Correlation analysis demonstrating the relationship between ECMscore and immune score, stromal score, ESTIMATE score, and tumor purity across six cohorts. (**B**) Heatmap illustrating normalized scores of immune cell populations in low- and high-ECMscore groups. Correlation plots (left) depict the associations between ECMscore and immune cell infiltrates. Yellow indicates positive or negative correlations, while grey represents no correlation. (**C**) Comparison of CD8 + T cells, M1 macrophages, fibroblasts, CYT, GEP, and IFN-γ levels between low- and high-ECMscore groups. (**D**) Bubble chart (upper) and Heatmap (lower) showcasing the correlation between immune modulators and ECMscore, along with their distributions in the low- and high-ECMscore groups

To discover the underlying relationship between the ECMscore and the immune landscape of HGSOC patients, a correlation analysis was conducted between the ECMscore and the immune score, ESTIMATE score, stromal score, and tumor purity. The results revealed that the ECMscore was negatively correlated with the immune score and ESTIMATE score, but positively correlated with tumor purity (Fig. 6A). Subsequently, the ssGSEA analysis indicated a significantly negative connection between ECMscore and immune infiltrate density in the TCGA-OV cohort (Fig. 6B). Specifically, the group with a high ECMscore had less infiltration of CD8 + T cells and M1 macrophages, and more fibroblasts, as confirmed by multiple algorithms (Fig. 6C and Supplementary Fig. S10A). Furthermore, we examined the status of CYT, GEP, and IFN-γ across low and high ECMscore groups. As shown in Fig. 6C, they were all elevated in the group with a low ECMscore, which was associated with a more immunoreactive microenvironment. Likewise, ECMscore was inversely correlated with immune modulators, and a low ECMscore was associated with an increased concentration of immune modulators (Fig. 6D). Moreover, the ECMscore signature displayed an adverse association with the cancer immunity cycle, which includes the release of cancer cell antigens (step 1), priming and activation (step 3), trafficking of immune cells to tumors (step 4), and killing of cancer cells (step 7) (Supplementary Fig. S10B).

To further investigate the association between ECMscore and immunological microenvironment, we evaluated the correlation between 14 genes and anticancer tumor-infiltrating immune cells (CD8 + T cells, NK cells, DCs, and macrophages) as well as CAFs. As a result, CXCL11, CXCL13, and LTA were significantly positively connected with CD8 + T cells, DCs, and macrophages, while MGP, COL8A2, and PAPPA were significantly positively correlated with CAFs (Supplementary Fig. S11A). Given the role of CAFs in interacting with cancer cells via growth factors, inflammatory ligands, and ECM proteins [7],we investigated the correlation between MGP, COL8A2, and PAPPA with CAF markers. The findings indicated a negative correlation between these genes and the CAF marker across different cohorts (Supplementary Fig. S11B). Additionally, we explored the detailed distribution of MGP, COL8A2, and PAPPA in ovarian cancer using single-cell RNA transcriptome data (GSE147082) from the TISCH database [54]. In comparison to other cell types, fibroblasts exhibited higher proportions of these genes, especially MGP (Supplementary Fig. S12A-D). A qRT-PCR experiment confirmed the positive association between MGP, COL8A2, PAPPA and FAP (an immunosuppressive macrophage marker) (Supplementary Fig. S12E).

### Pan-cancer exploration of the ECMscore signature

**Fig. 7:**
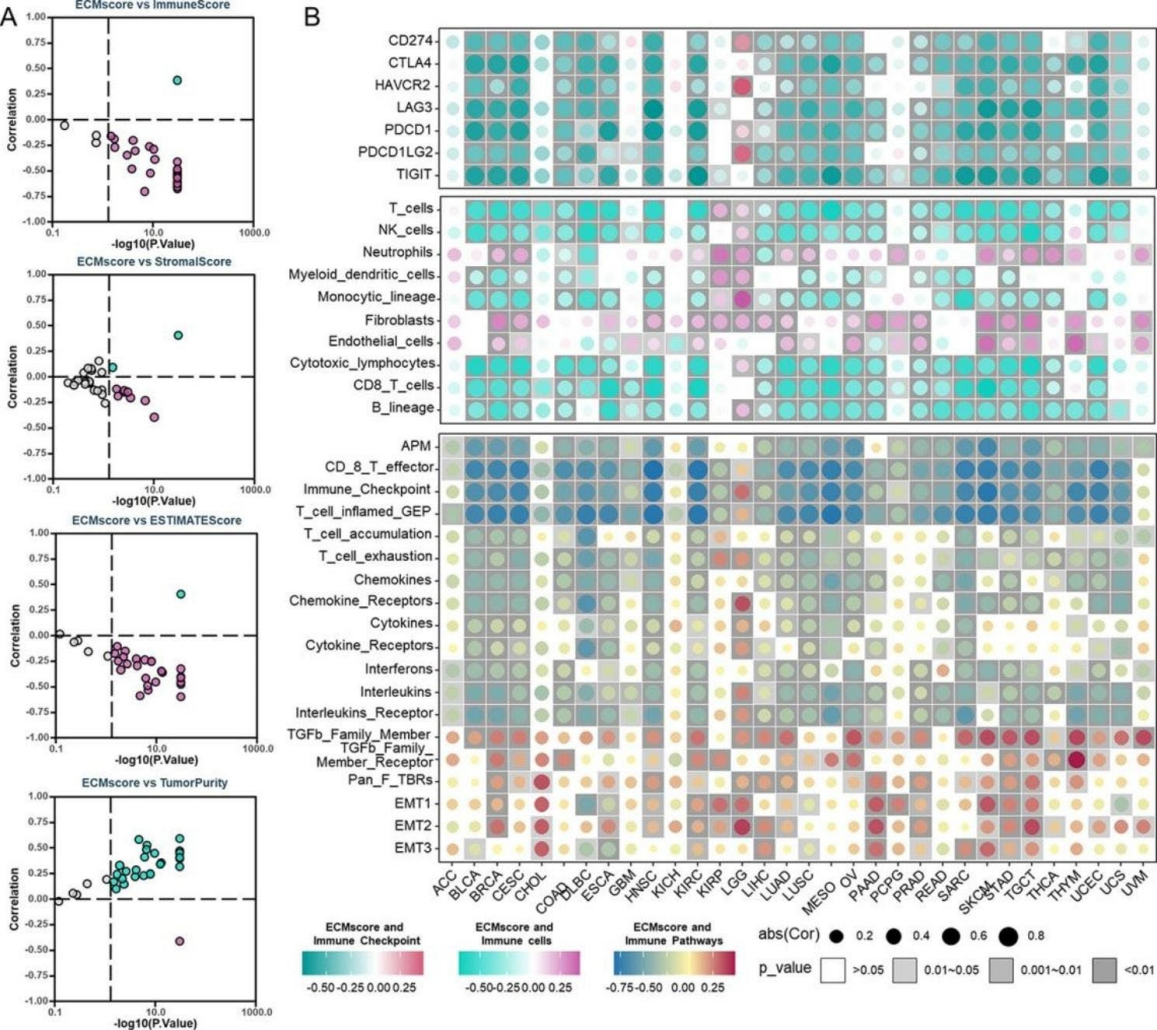
**Pan-cancer analyses of ECMscore signature.** (A) Correlation analysis demonstrating the relationships between ECMscore and immune score, stromal score, ESTIMATE score, and tumor purity in the pan-cancer cohort. Purple indicates negative correlation, while green indicates positive correlation. (B) Correlation analysis between ECMscore and immune checkpoints (upper), immune cell populations (middle), and TME signatures (lower)

To investigate the pan-cancer TME landscape influenced by the ECMscore signature, we analyzed the relationship between ECMscore, immune score, and stromal score. Remarkably, the ECMscore exhibited consistent negative associations with immune score, ESTIMATE score, and stromal score, while showcasing a positive correlation with tumor purity across multiple cancer types (Fig. 7A). Moreover, a closer examination revealed a negative correlation between ECMscore and the expression of critical immune checkpoints as well as major immune cells in most cancers (Fig. 7B). Furthermore, substantial stromal component cells like fibroblasts and endothelial cells displayed correlations with the ECMscore (Fig. 7B). Notably, the ECMscore demonstrated a negative correlation with immune activation and a positive correlation with stromal activation and EMT activation (Fig. 7B). These collective findings strongly suggest that the ECMscore holds the potential to serve as a reflection of the immune landscape of pan-cancer.

### Impact of ECMscore on immunotherapy and chemotherapy strategies

**Fig. 8:**
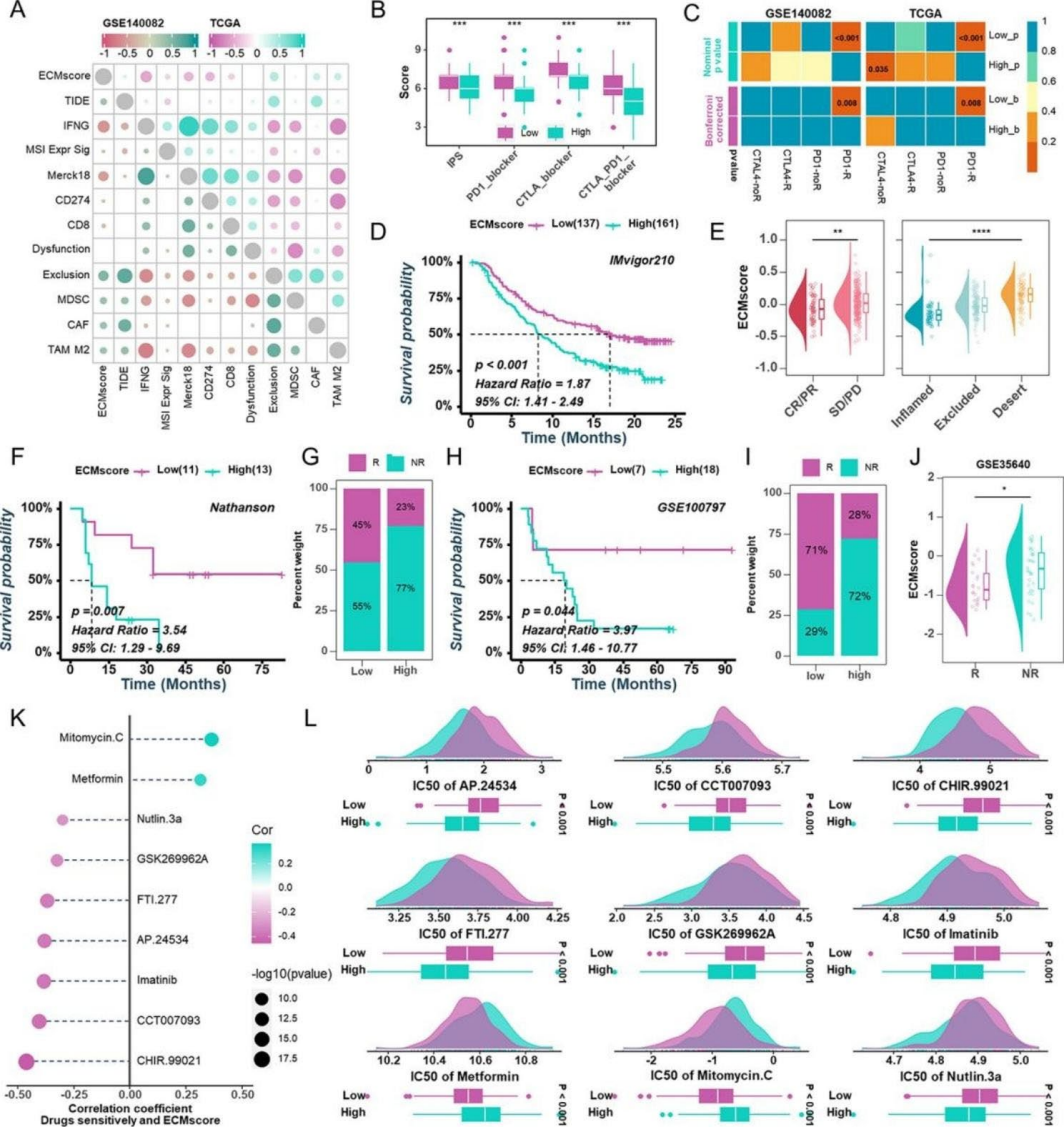
**Implications of ECMscore for immunotherapy and chemotherapy.** (**A**) Correlation analysis between ECMscore and TIDE scores in GSE140082 and TCGA-OV cohorts. (**B**) Comparison of IPS between low- and high-ECMscore groups stratified by PD-1 and CTLA4 expression. (**C**) Relationship between ECMscore groups and immunotherapy responses using the TIDE algorithm. (**D**) Kaplan-Meier analysis estimating OS in different ECMscore groups in the IMvigor cohort. (**E**) Distribution of ECMscore in patients with distinct immunotherapy responses (left) and immunophenotypes (right) in the IMvigor cohort. (**F**) Kaplan-Meier analysis estimating OS in different ECMscore groups in Nathanson’s cohort. (**G**) Distribution of ECMscore in patients with varying immunotherapy responses in Nathanson’s cohort. (H) Kaplan-Meier analysis estimating OS in different ECMscore groups in the GSE100797 cohort. (**I**, **J**) Distribution of ECMscore in patients with diverse immunotherapy responses in the GSE100797(**I**) and GSE35640 cohort. (**K**) Correlation analysis between ECMscore and IC_50_ values of candidate drugs in TCGA-OV cohort. (**L**) Comparison of IC_50_ values of candidate drugs between low- and high-ECMscore groups in TCGA-OV cohort

Considering that the group with a low ECMscore had a stronger immune response potential and maintained enhanced immune checkpoint expression, we investigated whether the ECMscore could predict HGSOC patient responses to immune checkpoint inhibitors. Using the TIDE method, we discovered that ECMscore exhibited a favorable association with TIDE, exclusion score, CAFs, and M2 macrophages, although no correlation with dysfunction score was observed in both the GSE140082 and TCGA-OV cohorts (Fig. 8A). Our investigation extended further to examine whether the low ECMscore group could be more responsive to immunotherapy. Utilizing the IPS and submap algorithm, consistent results were obtained, reinforcing the notion that patients with a low ECMscore might be more prone to positive immunotherapy outcomes (Fig. 8B C). Expanding our analysis to various immunotherapy datasets, including IMvigor (urothelial cancer), Nathanson’s cohort (melanoma), GSE100797 (melanoma), and GSE35640 (melanoma), we found that individuals with a high ECMscore were associated with an immunological desert phenotype, poorer prognosis, and weaker immune response (Fig. 8D J). Intriguingly, by calculating the half maximum inhibitory concentration (IC_50_) value for various drugs using pRRophetic software, we established a connection between ECMscore and drug sensitivity in HGSOC samples (Fig. 8K L). Nine medications emerged from this analysis, with the IC_50_ of two compounds positively correlated with ECMscore, while the IC_50_ of seven other drugs demonstrated a negative correlation. Notably, the low ECMscore group appeared to be more susceptible to cisplatin, etoposide, and paclitaxel (Supplementary Fig. S13A). Collectively, these results suggested that the ECMscore may be a valuable biomarker in guiding HGSOC patients towards optimal treatment strategies.

## Discussion

It is known that the malignancy of HGSOC is considerable, and that the majority of patients with advanced HGSOC have a dismal prognosis, with a 5-year survival rate of approximately 30% [4]. However, not every patient experiences a relapse, indicating the heterogeneity within this cancer subtype. In 2011, the cancer genome atlas (TCGA) evaluated mRNA expression, miRNA expression, DNA copy number, and promoter methylation in 489 advanced serous cancers, as well as DNA sequences encoding gene exons in 316 of these tumors. This is a significant step forward in comprehending HGSOC [55]. This diversity highlights the need for a stratified approach to patient management, making the development of effective biomarkers and targeted therapies critical. ECMs play a pivotal role in the TME, influencing tumor behavior and metastatic potential. Understanding the role of ECMs in HGSOC is essential for the advancement of both biomarker discovery and novel therapeutic strategies [17, 56]. To enhance prognostic prediction and treatment selection, this study proposes a classification strategy centered around ECM genes, resulting in the establishment of a 14-gene signature (MGP, COL8A2, PAPPA, NYX, PLXNA1, CST6, LOXL4, SERPINA10, TGM7, CXCL11, CXCL13, HCFC2, LTA, WNT9A) specific to HGSOC. By utilizing the ECMscore derived from this signature, patients with HGSOC are segregated into two distinct groups. Through rigorous Cox regression analyses, both univariate and multivariate, the ECMscore emerges as an independent prognostic factor. Remarkably, the utility of ECMscore extends beyond HGSOC, proving its value as a prognostic marker across a diverse range of cancers including bladder urothelial carcinoma, colon adenocarcinoma, and pancreatic adenocarcinoma, among others. This underlines its potential as a versatile biomarker across various malignancies. Furthermore, the relationship between this model and the biological behavior, genetic characteristics, and immunological landscape of HGSOC was thoroughly studied, and these results were validated on a pan-cancer scale. After investigating the involvement of ECMscore in the development of HGSOC, the study provides valuable insights into the formulation of more targeted therapeutic strategies.

ECM participates in both the modulation and the therapy of tumors, and has shown great value in both the diagnosis and the prognosis of cancer [57–59]. Among the ECM genes that were identified as prognostically significant in this study, many were enriched in pathways related to ECM organization, structural components, collagen-containing ECM, and cytokine-cytokine receptor interactions. For instance, MGP, an ECM protein, has been implicated in carcinogenesis and its dysregulated expression observed in multiple tumor types. Targeting MGP has demonstrated potential in reducing the growth of colorectal cancer tumors and reversing resistance to certain chemotherapies [13]. Similarly, CXCL11 has been linked to migration and metastasis promotion in hepatocellular carcinoma [60], while also showing immune-related and better prognostic implications in colon cancer [14]. LOXL4’s deletion has been found to enhance tumor growth and metastasis in triple-negative breast cancer through ECM-related mechanisms [61]. PAPPA, on the other hand, demonstrates dual roles, with its inhibition leading to decreased ovarian cancer cell growth, invasion, and metastasis, while overexpression can increase tumor growth [15, 16]. Studies have also indicated that the ECM-receptor interaction pathway is filled with potential indicators of HGSOC metastasis [62]. Consistent with previous research, we discovered that ECMscore was related to malignant signaling pathway activity. The high ECMscore group showed a positive correlation with stromal and carcinogenic pathways, while the low ECMscore group exhibited enrichment in immune-related pathways. Nonetheless, association study with genetic characteristics reveals that ECMscore has no effect on this variable. Although genetic instability is a feature of HGSOC, ECM may impact the cellular microenvironment and can directly influence prognosis and chemoradiotherapy response [16]. In general, the role of ECM in tumors is closely tied to its modulation of immune regulation [63].

We further thoroughly investigated the intricate relationship between the immunological profile of HGSOC patients and the ECMscore. The ssGSEA analysis unveiled a strong negative correlation between ECMscore and immune infiltrate density, revealing that patients with high ECMscores had reduced CD8 + T cell and M1 macrophage infiltration but elevated fibroblast presence. These findings correspond to the conclusion that the high ECMscore group exhibited a less immunoreactive microenvironment, which was in line with their poorer prognosis. CD8 + intraepithelial tumor-infiltrating lymphocytes are associated with a favorable prognosis in HGSOC, as their presence indicates heightened OS and progression-free survival [64, 65]. The diminished CD8 + T cell and M1 macrophage infiltration in the high ECMscore group may contribute to their worse prognosis. This interaction with the immune microenvironment underscores the significance of the ECMscore in predicting the immune response and its clinical implications. CAFs, which are crucial stromal compartment components, interact with cancer cells by secreting cytokines and growth factors as well as ECM proteins. CAFs can influence immune cell infiltration, drug delivery, and immune evasion, affecting cancer progression and therapy response [66–68]. In our study, we discovered a highly positive association between MGP, COL8A2, PAPPA, and CAFs. It has been demonstrated that MGP, COL8A2, and PAPPA are effective immunological checkpoints that mediate tumor immunosuppression [26, 69–72]. Among these, PAPPA was discovered to be a highly differentially expressed therapeutic target in Ewing sarcoma [26], COL8A2 is a crucial part of the basement membrane of corneal endothelial cells, and it can encourage the malignant development of glioblastoma cells by triggering EMT [73]. Notably, MGP has shown potential as a novel and significant mediator for mesenchymal stem cell-mediated immunoregulatory treatment in Crohn’s disease, as indicated by recent research [25]. Further validation was provided through qRT-PCR tests, confirming that the set of 14 prognostic-associated genes, particularly MGP, COL8A2, and PAPPA, are correlated with FAP expression - an established marker of immunosuppressive macrophages. Moreover, a comprehensive pan-cancer analysis lent support to these findings by revealing that the ECMscore potentially reflects the immune landscape across various cancer types. The implications of these findings emphasize the need for expanded investigations into different cancer contexts.

In addition to investigating networks and mechanisms, our study delved into the predictive capacity of ECMscore for immunotherapy response in cancer patients. The outcomes strongly indicate that individuals with a low ECMscore are more likely to exhibit a favorable response to immunotherapy. This observation was reinforced by the analysis of multiple immunotherapy datasets, encompassing urothelial cancer and melanoma. Conversely, individuals with a high ECMscore exhibited a higher proportion of an immunological desert phenotype, associated with a poorer prognosis and diminished immune response. Furthermore, we explored the potential of ECMscore to predict chemotherapeutic sensitivity by assessing its correlation with the IC_50_ values of different drugs in HGSOC patients. Encouragingly, our investigation unveiled that cisplatin, etoposide, and paclitaxel demonstrated increased sensitivity in patients with a low ECMscore. Additionally, alternative mechanisms need to be further studied by doing combination analysis using the ECM-related risk signature, aside from those pathways elucidated in this study.

## Conclusion

In summary, we established a 14-gene risk profile based on ECM that may accurately predict the survival outcomes and response to immunotherapy and chemotherapy in patients with HGSOC and pan-cancer based on a comprehensive analysis based on large-scale clinical samples and transcriptome data. These discoveries have not yet been verified in human models of HGSOC. However, these results hold promise for emerging as a robust prognostic instrument with future implications for prognostication and determination of chemotherapeutic efficacy, not only within the context of HGSOC but also in the broader realm of pan-cancer research.

### Electronic supplementary material

Below is the link to the electronic supplementary material.

Supplementary Material 1

Supplementary Material 2

Supplementary Material 3

Supplementary Material 4

Supplementary Material 5

Supplementary Material 6

Supplementary Material 7

## Data Availability

The datasets that support the findings of this study are available from the corresponding author, [X.F.], upon reasonable request.
