# Supplementary material for: Integrative evaluation and experimental validation of the immune-modulating potential of dysregulated extracellular matrix genes in high-grade serous ovarian cancer prognosis
Source: Cancer Cell Int. 2023 Sep 30;23:223. doi: 10.1186/s12935-023-03061-y (PMC10543838; doi:10.1186/s12935-023-03061-y)
Supplement: Supplementary file 1 — Supplementary Material 1 [file 12935_2023_3061_MOESM1_ESM.docx]

**Supplementary Figures**

**
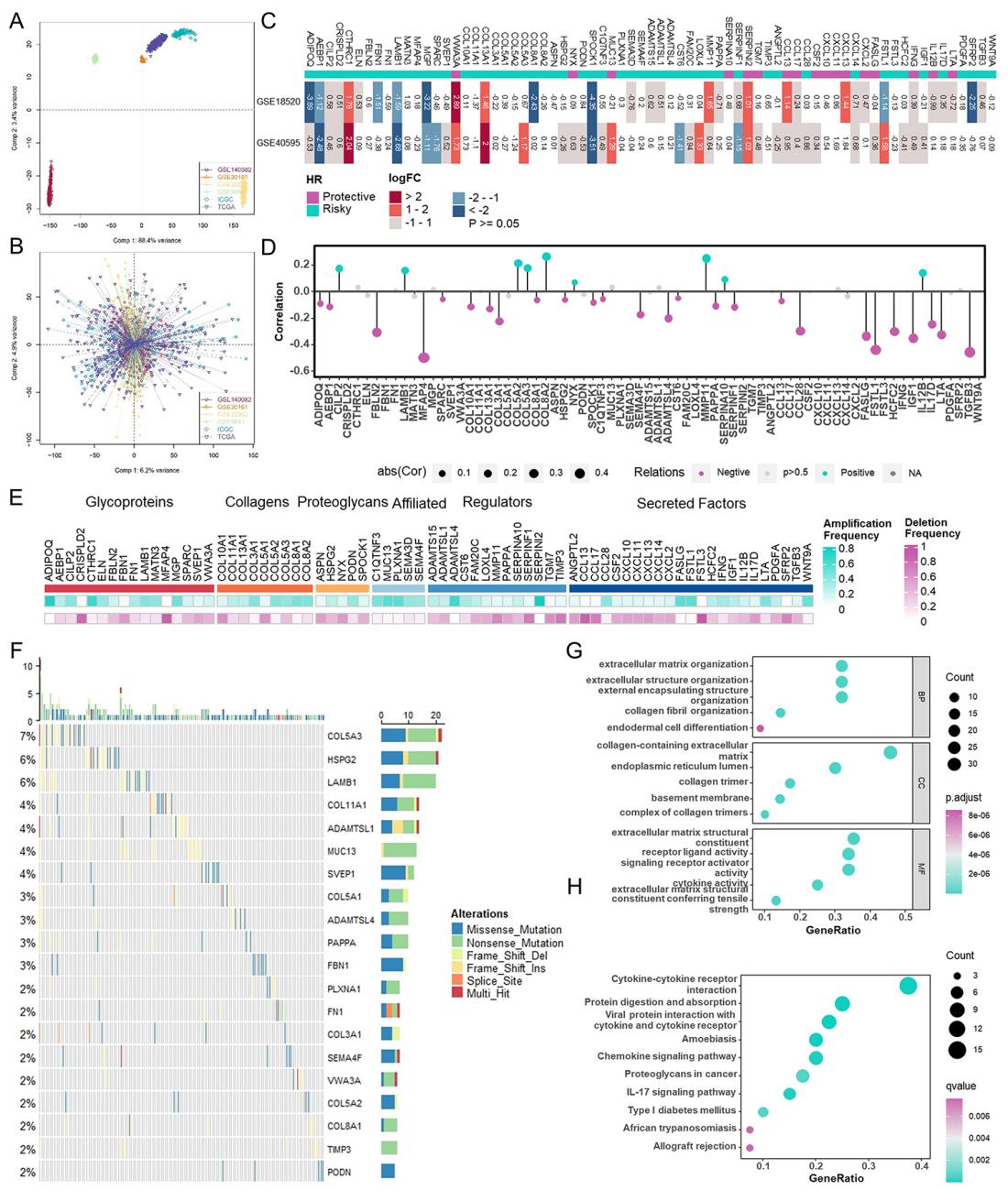
**

**Fig. S1: Selection of prognostic-related ECM genes in HGSOC patients.**

(A, B) Principal component analysis illustrating gene expression distribution in four GEO cohorts, ICGC-OV, and TCGA-OV datasets before (A) and after (B) batch effect correction.

(C) Expression of prognostic-related ECM genes in GSE18520 and GSE40595.

(D) Correlation between the expression levels and methylation of prognostic-related ECM genes.

(E)Heatmap displaying the amplification frequency (upper) and deletion frequency (bottom) of prognostic-related ECM genes.

(F) Waterfall plot showcasing prognostic-related ECM genes in the TCGA-OV cohort.

(G, H) GO (G) and KEGG（H) enrichment analysis based on prognostic-related ECM genes.

**
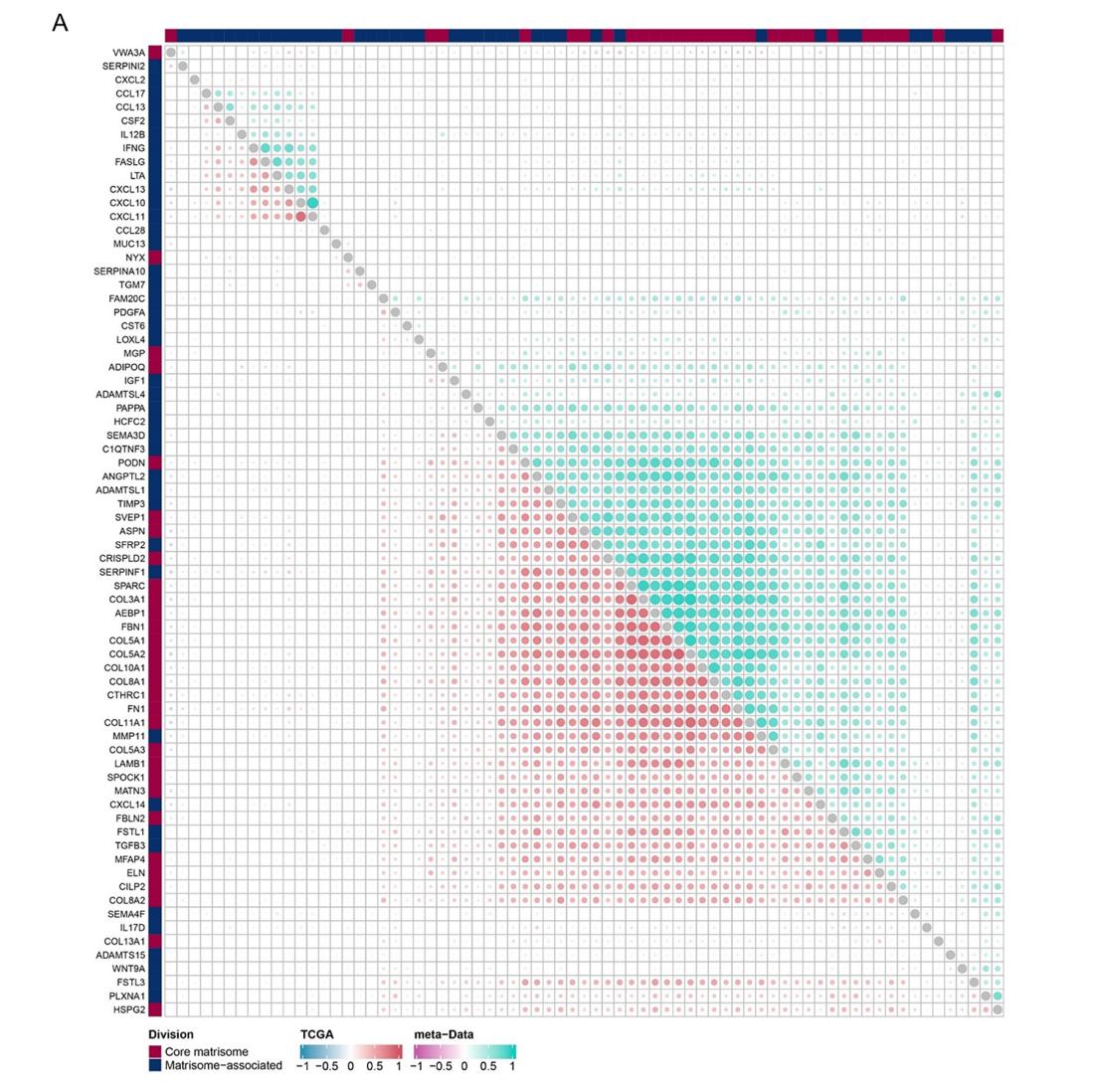
**

**Fig. S2: Correlation analysis between prognostic-related ECM genes in meta-data and TCGA-OV cohort.**

**
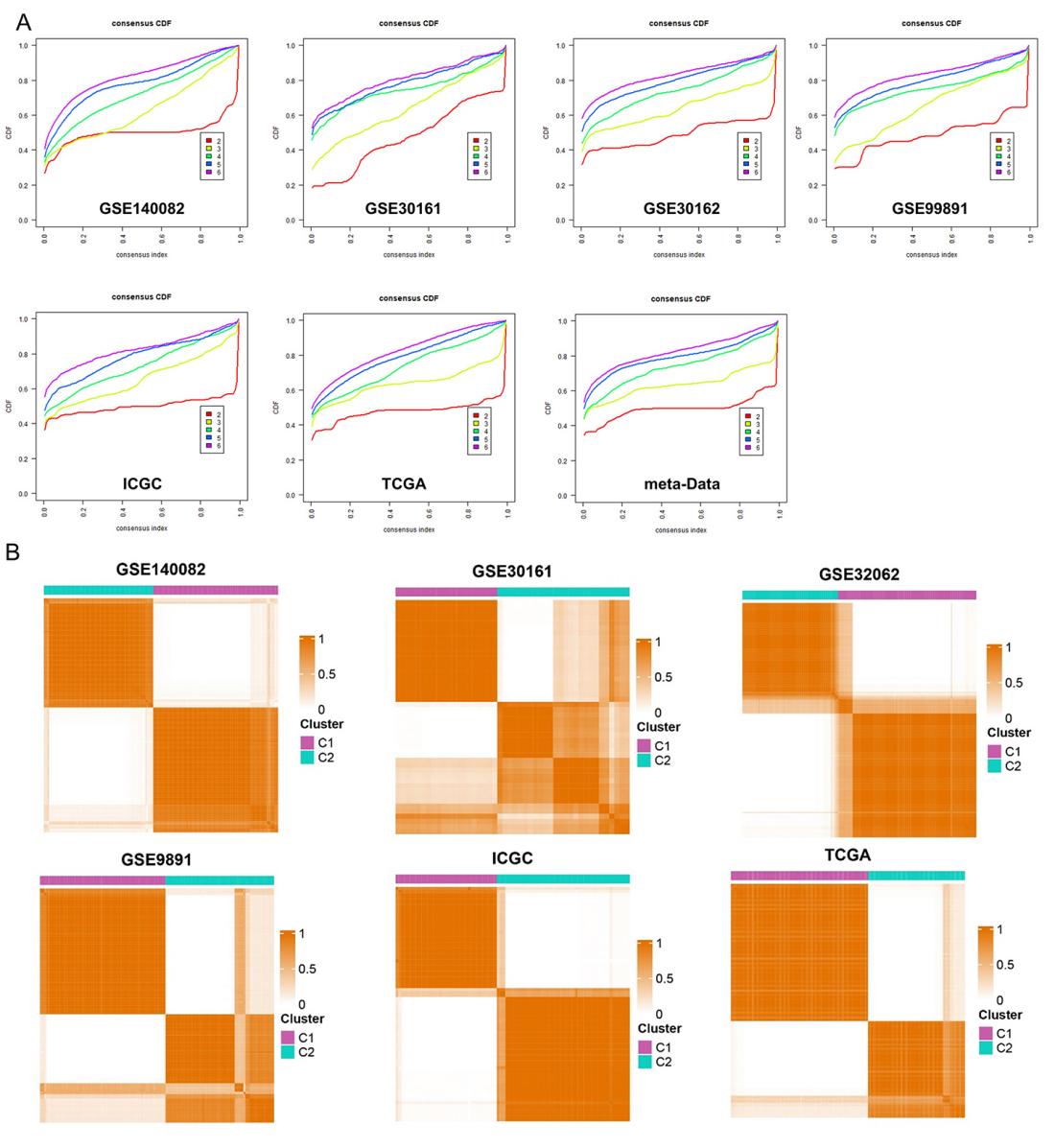
**

**Fig. S3: Identification of two ECM clusters in different cohorts.**

(A) Cumulative distribution function curve depicting consensus distributions for each k value (ranging from 2 to 6) in different cohorts.

(B) Consensus clustering cumulative distribution function for k = 2 in different cohorts.

**
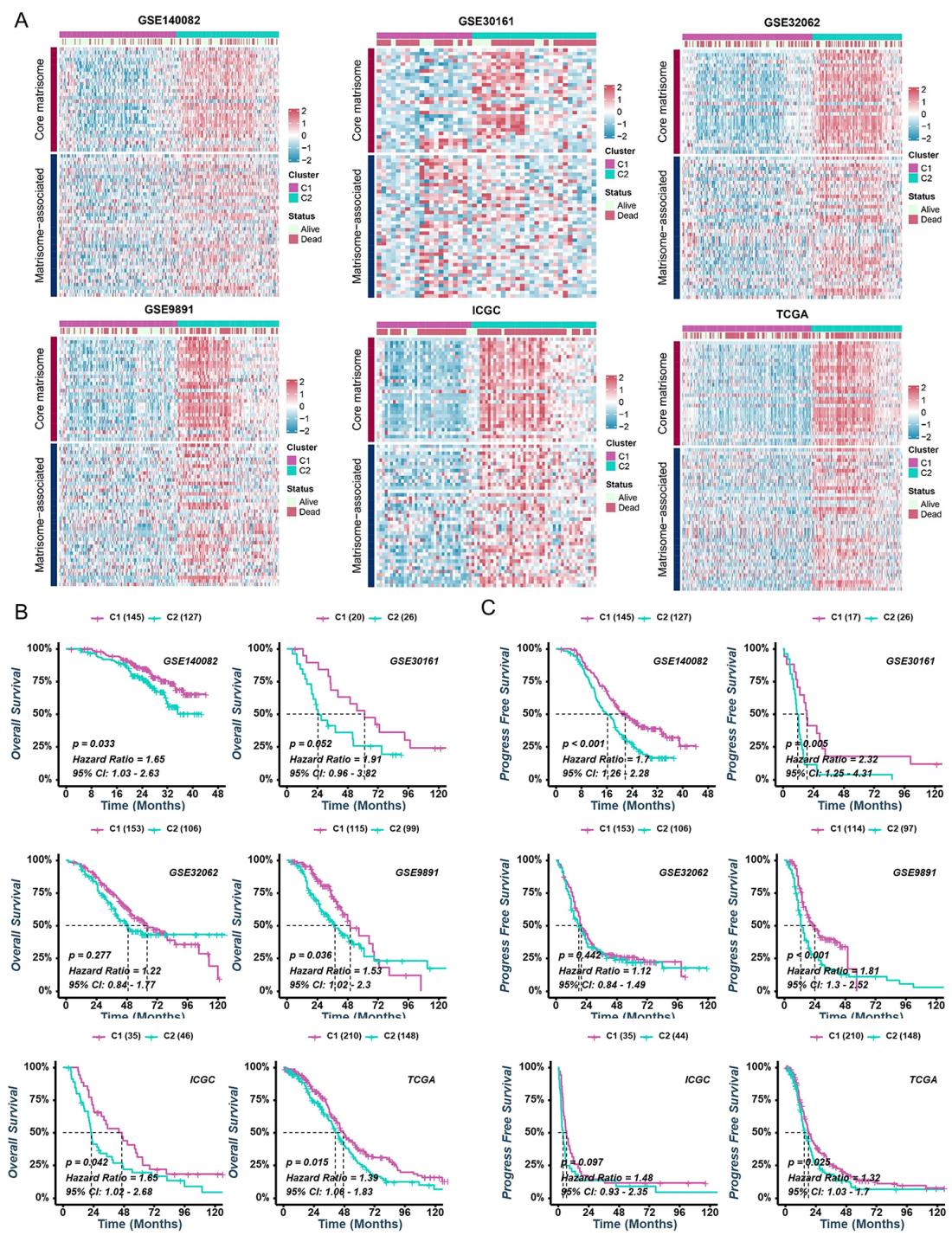
**

**Fig. S4: Unsupervised consensus clustering analyses identified two ECM clusters with heterogeneous prognosis.**

(A) Heatmap presenting the transcriptome profiles of the prognostic-related ECM genes in various cohorts.

(B, C) Kaplan-Meier analyses estimating OS (B) and PFS (C) between cluster C1 and cluster C2 in different cohorts.

**
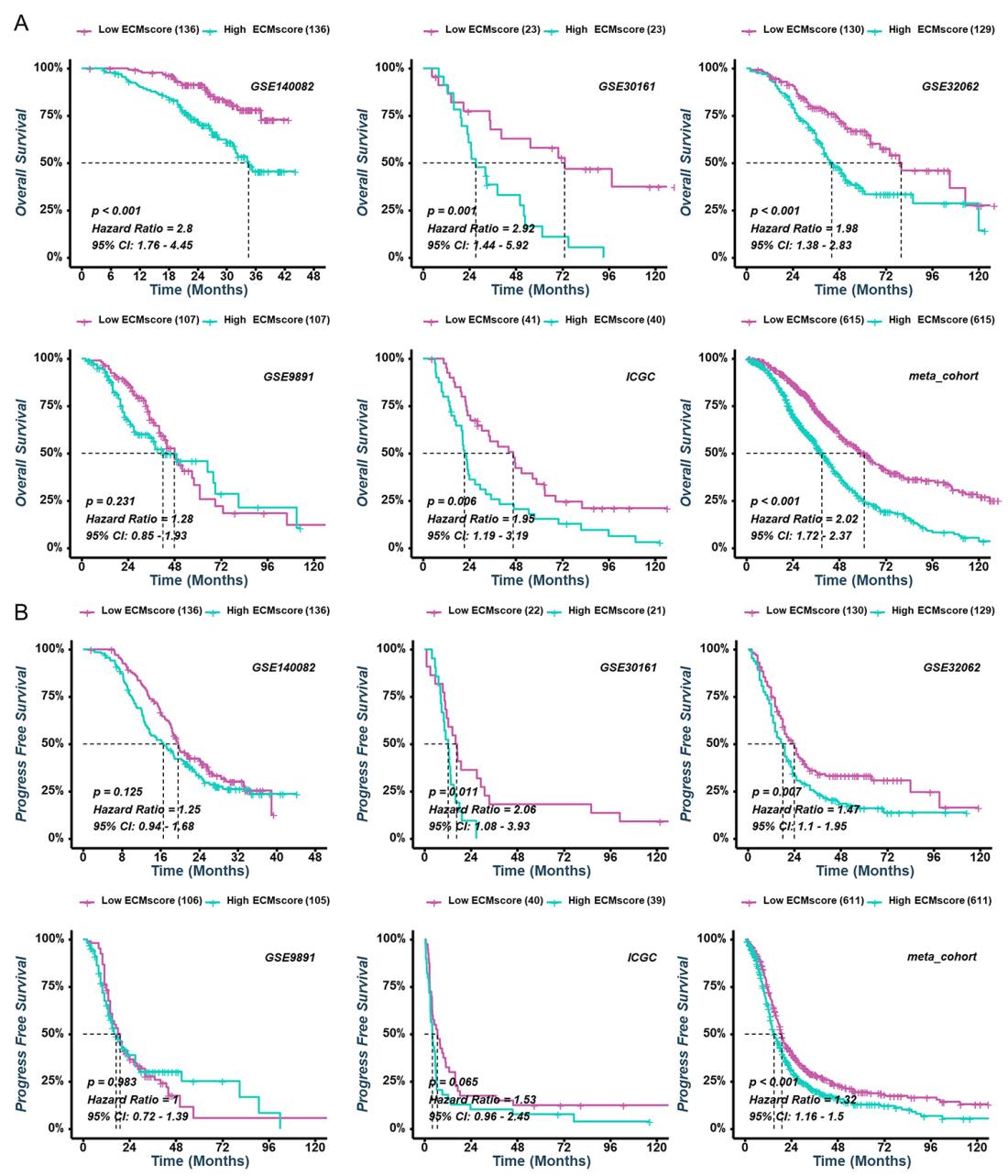
**

**Fig. S5: Validation of the ECMscore in six independent cohorts.**

(A, B) Kaplan-Meier analyses estimating OS (A) and PFS (B) between low- and high-ECMscore groups in various cohorts.

**
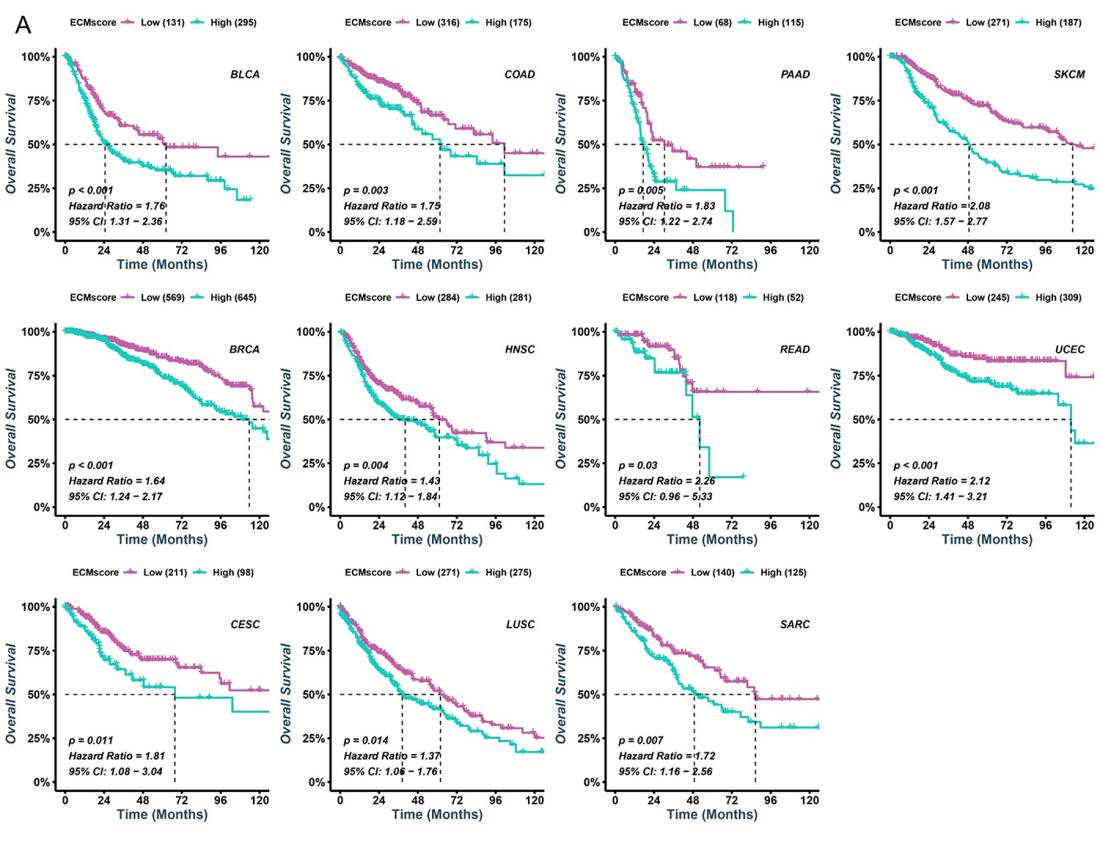
**

**Fig. S6: Kaplan-Meier analyses estimating OS between low- and high-ECMscore groups in pan-cancer cohorts. The "survminer" package was used to split the HGSOC patients into low and high ECMscore groups based on the optimal cutoff value.**

**
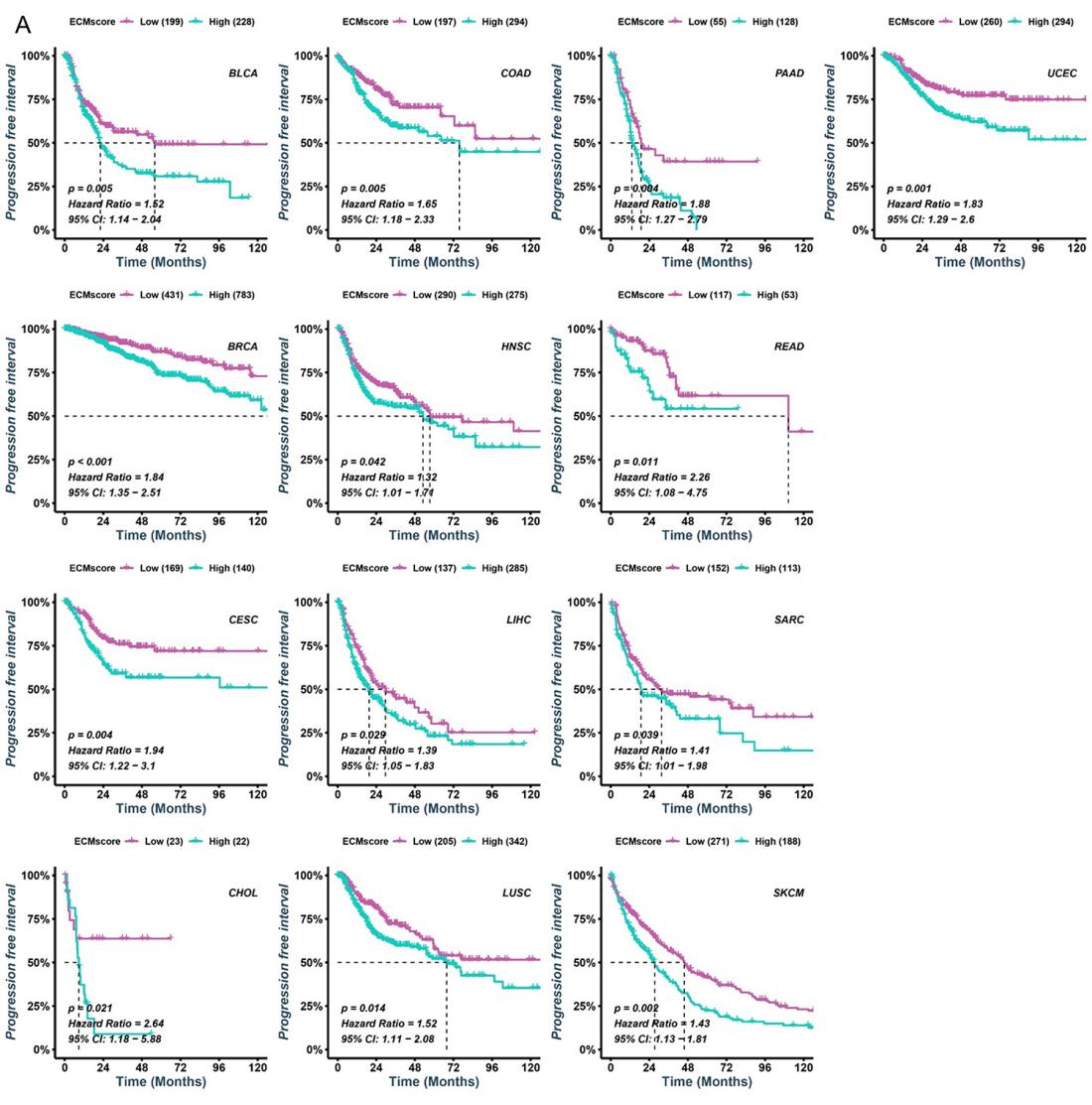
**

**Fig. S7: Kaplan-Meier analyses estimating PFS between low- and high-ECMscore groups in pan-cancer cohorts.**

**
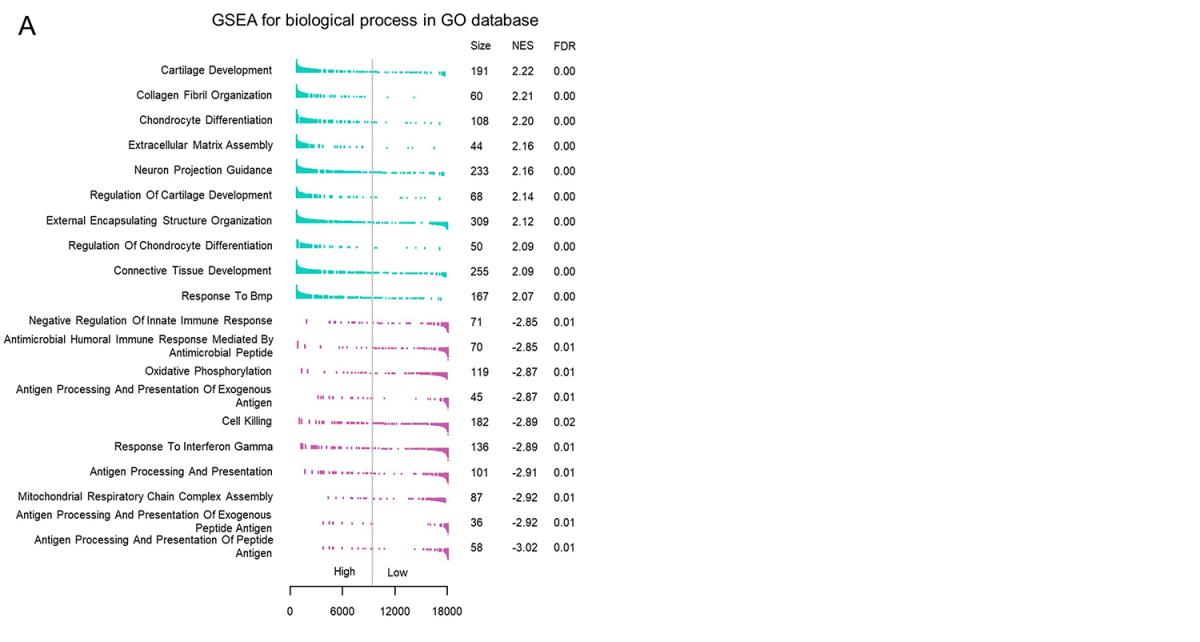
**

**Fig. S8: Underlying biological functions of different ECMscore groups.**

(A)GSEA results of biological process gene sets from the GO database and the top 10 positively and negatively related pathways with ECMscore were presented.

**
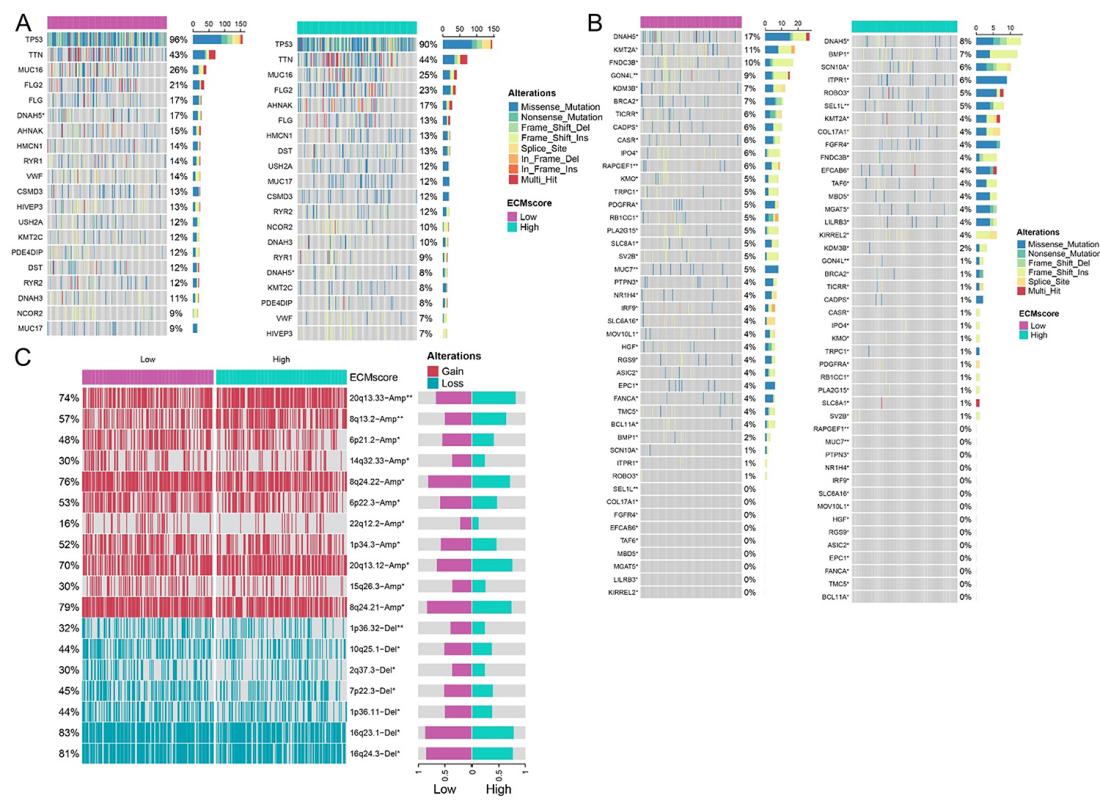
**

**Fig. S9: Genomic Features of different ECMscore groups.**

(A) Waterfall plot illustrating the top 20 most frequently mutated genes in the TCGA-OV cohort.

(B) Waterfall plot highlighting genes with differing mutation frequencies between the low- and high-ECMscore groups.

(C) Heatmap (left) depicting the broad-level copy number alterations in the low- and high-ECMscore groups. Bar chart (right) presenting the frequency of amplifications or deletions in the low- and high-ECMscore groups.

**
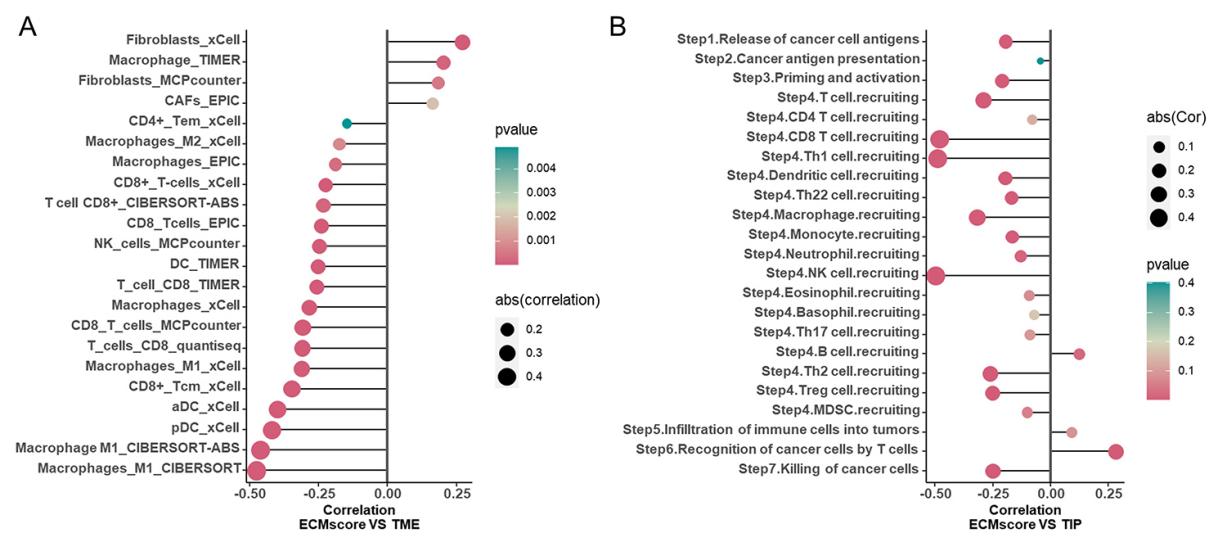
**

**Fig. S10: The association between the ECMscore and immune landscape.**

(A) Correlation between ECMscore and various anticancer tumor-infiltrating immune cells using diverse algorithms.

(B) Correlation between ECMscore and the cancer immunity cycle in the TCGA-OV cohort.

**
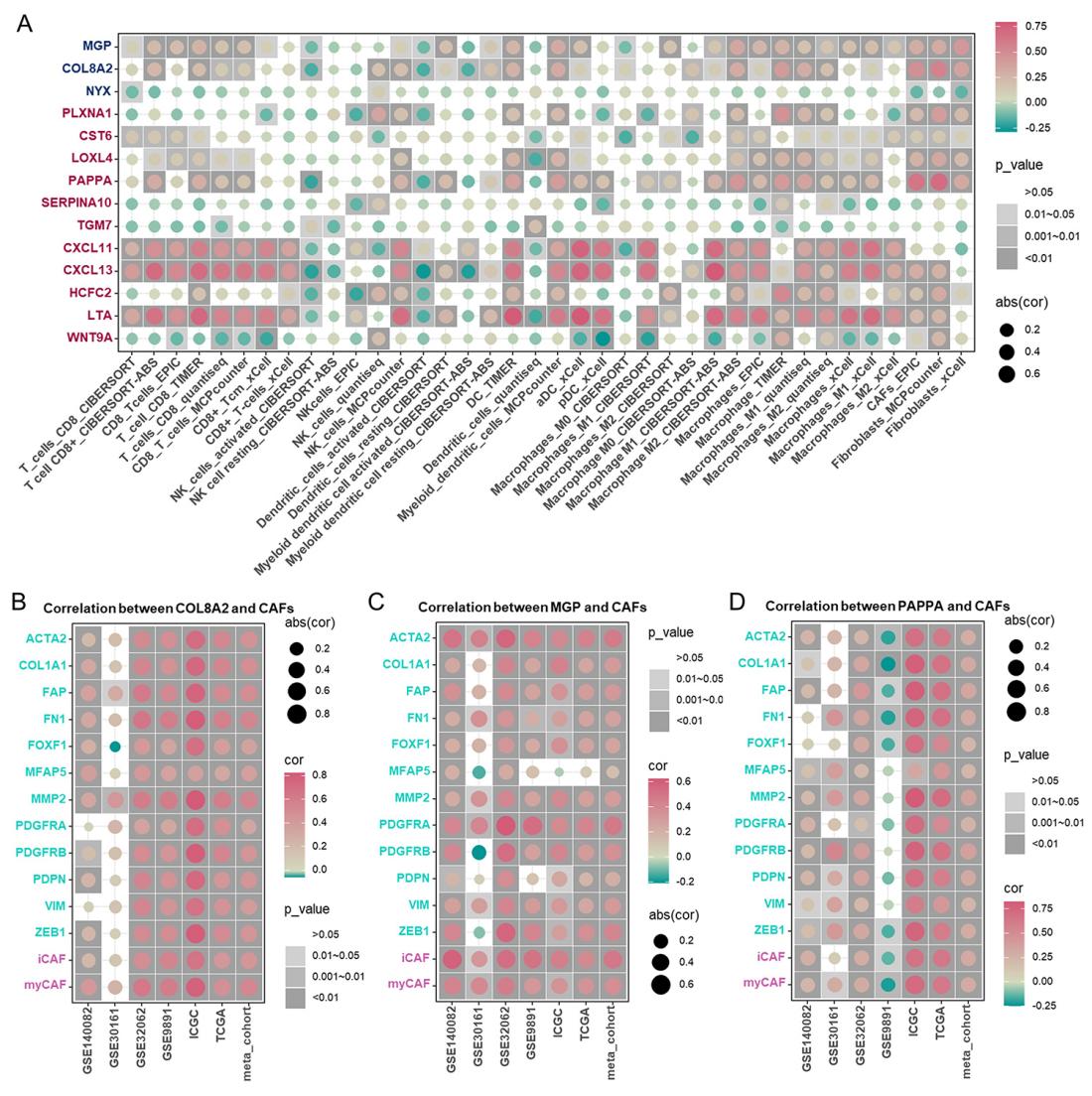
**

**Fig. S11: The association between the ECM genes and immune landscape.**

(A) The correlation between the expression of 14 prognostic genes and various anticancer tumor-infiltrating immune cells. The color and size of the points reflect the correlation. The red color on the y-axis labels represents matrisome-associated genes, while the blue color represents core matrisome genes.

(B-D) The correlation between the expression of CAFs markers and COL8A2 (B), MGP (C), and PAPPA (D). The size and color of the points correspond to the correlation. The green color on the y-axis labels represent CAFs markers, while the red color represent iCAF and myCAF scores obtained through the GSVA algorithm.

**
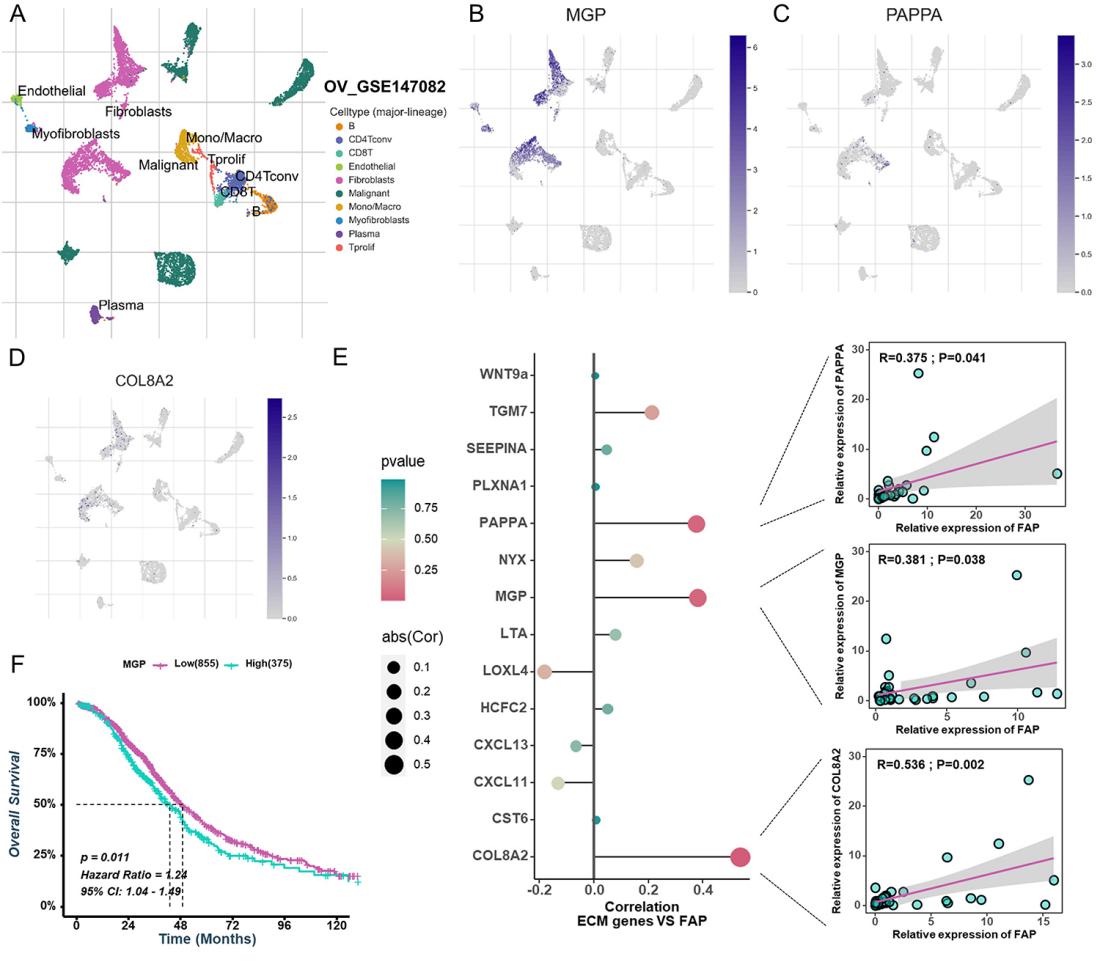
**

**Fig. S12: Validation of COL8A2, MGP, and PAPPA in single-cell RNA transcriptome data.**

(A-D) Expression patterns of COL8A2, MGP, and PAPPA across various cell types in the GSE147082 dataset. Cell clustering and annotation were performed based on the TISCH databases.

(E) Validation of the correlation between 14 ECM-related genes and FAP using qRT-PCR in the Xiangya cohort.

**
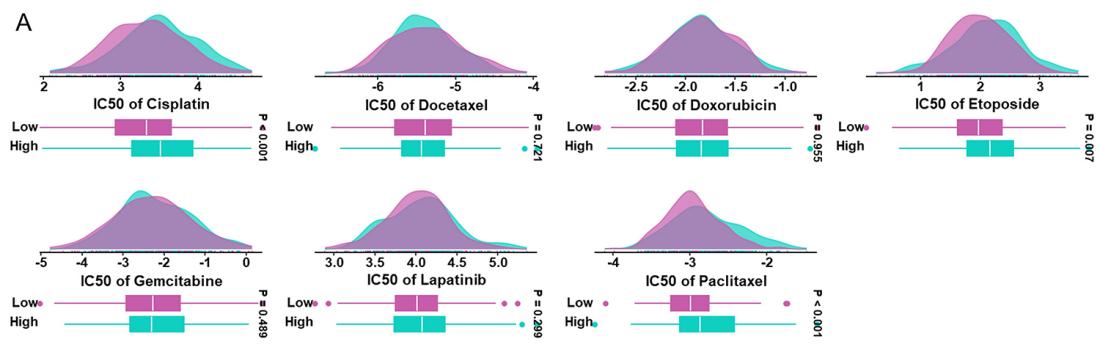
**

**Fig. S13: The IC50 values of several common chemotherapy drugs between low- and high-ECMscore groups in the TCGA-OV cohort.**
